# Supplementary material for: Single-cell transcriptomes of pancreatic preinvasive lesions and cancer reveal acinar metaplastic cells’ heterogeneity
Source: Nat Commun. 2020 Sep 9;11:4516. doi: 10.1038/s41467-020-18207-z (PMC7481797; doi:10.1038/s41467-020-18207-z)
Supplement: Supplementary file 1 — Supplementary Information [file 41467_2020_18207_MOESM1_ESM.pdf]

Supplementary Information

Single cell transcriptomes of pancreatic pre-invasive lesions and cancer reveal acinar metaplastic cells' heterogeneity

Schlesinger *et al.*

Supplementary Figures 1-11

Supplementary Fig. 1

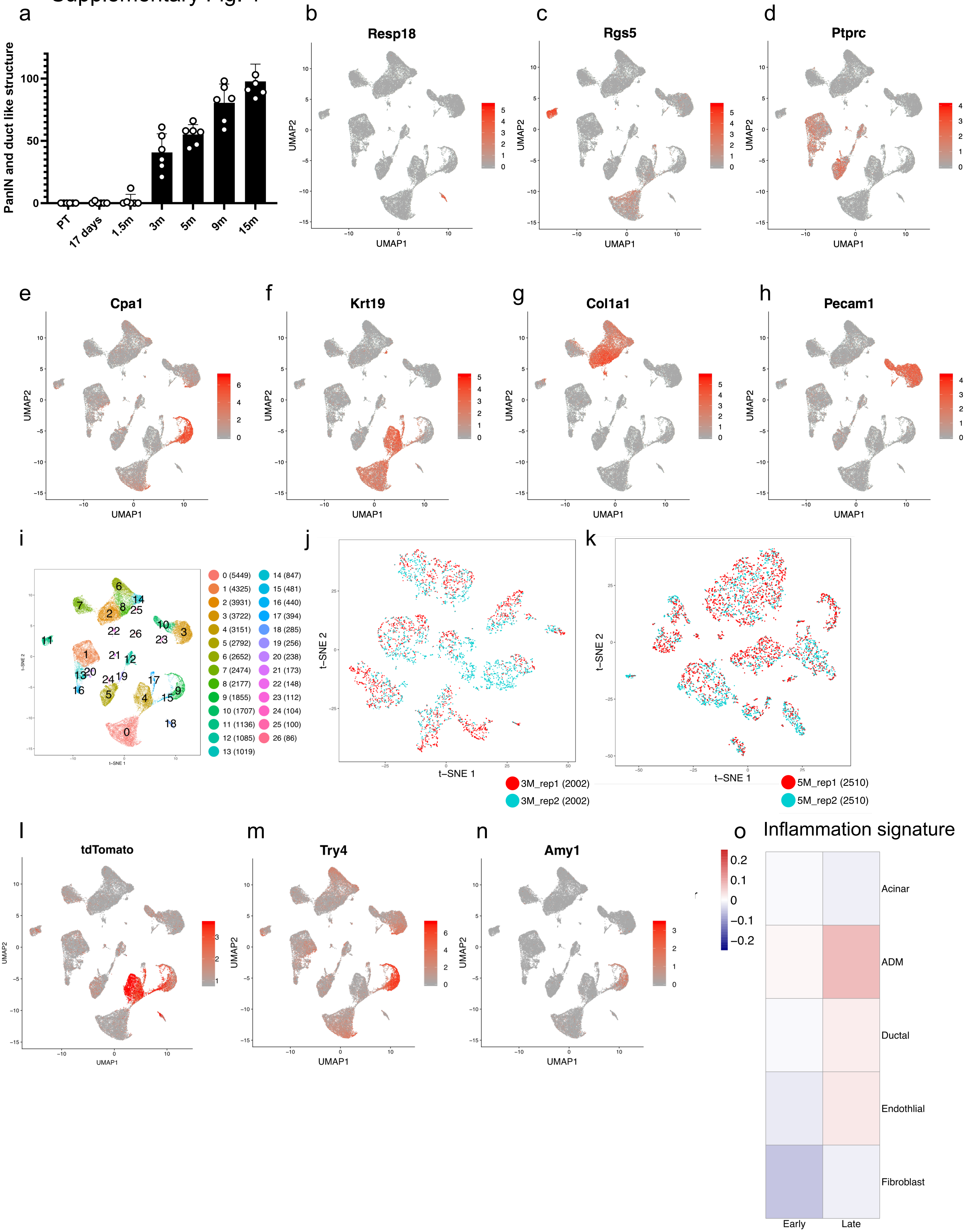

Supplementary Fig. 1: Characterization of pancreatic lesions infiltrating cells.

(a) Number of PanINs and duct-like structures. X-axis represents time points post tamoxifen injection (PTI). On the Y-axis, total numbers of PanINs and duct-like structures are indicated. To measure the number of PanINs, Krt19-stained slides were photographed, and the total number of lesions in the surface areas of six randomly selected fields were counted. Bars show mean values +/- SD.

(b-h) UMAP showing the expression level of representative markers for each cell type: (b) *Resp18*-neuroendocrine cells, (c) *Rgs5*-pericytes, (d) *Ptprc (CD45)*-immune cells, (e) *Cpa1*-acinar cells, (f) *Krt19*-ductal cells, metaplastic and tumor cells, (g) *Col1a1*-fibroblasts, (h) *Pecam1* (*CD31*)-endothelial cells. High expression level - red, low expression level - gray. Scale is on the right and relevant gene name on the top of each chart.

(i) UMAP of all cells, cluster numbers are shown.

(j-k) tSNE showing cells from the two biological replicates of three months PTI samples (j) and five months PTI samples (k). The cells from each of the two replicates were equally distributed with minimal batch effects. To have the same number of cells from each repeat, we randomly picked cells from the sample that included a larger number of cells.

(l-n) UMAP showing the expression level of selected genes that are expected to be expressed in acinar cells: (l) *tdTomato*, (m) *Try4*, (n) *Amy1*. High expression level - red, low expression level - gray. Scale is on the right and relevant gene name on the top of each chart.

(o) Heatmap showing inflammation score. ADM refers to late metaplastic cells (*tdTomato*-positive, *Cpa1*-negative). X axis- early and late time points. Y axis - cell types as indicated in Fig. 1i. Scale is shown on the left.

Supplementary Fig. 2

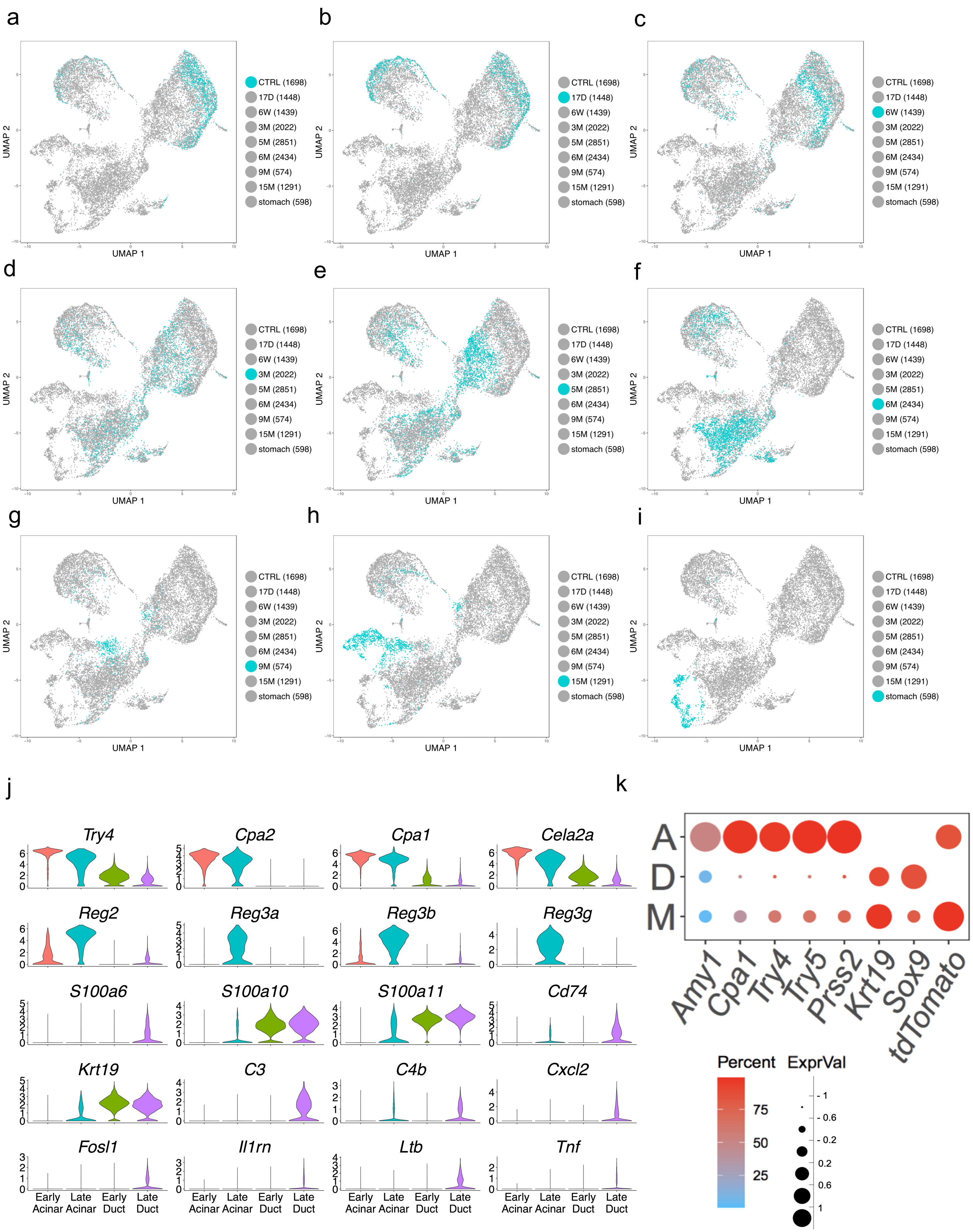

Supplementary Fig. 2: Acinar and ductal cells - changes in transcription profile post tamoxifen injection.

(a-i) In each UMAP chart, cells from one time point are colored in turquoise and all other cells in grey. In (i) stomach epithelial cells are highlighted.

(j) Violin plot representing the expression level of selected genes that are differentially expressed at early and late time points PTI. Expression levels on the Y axis - normalized expression.

(k) Dot plot showing the expression level and abundance of acinar related enzymes, acinar and ductal markers. A - includes all acinar cluster cells (*Cpa1*-Positive), D - includes all ductal cluster cells (*Krt19*-positive, *tdTomato* negative cells), M - includes all metaplastic cluster cells (*tdTomato* – positive, Cpa1-negative). Gene names are shown at the bottom.

Supplementary Fig. 3

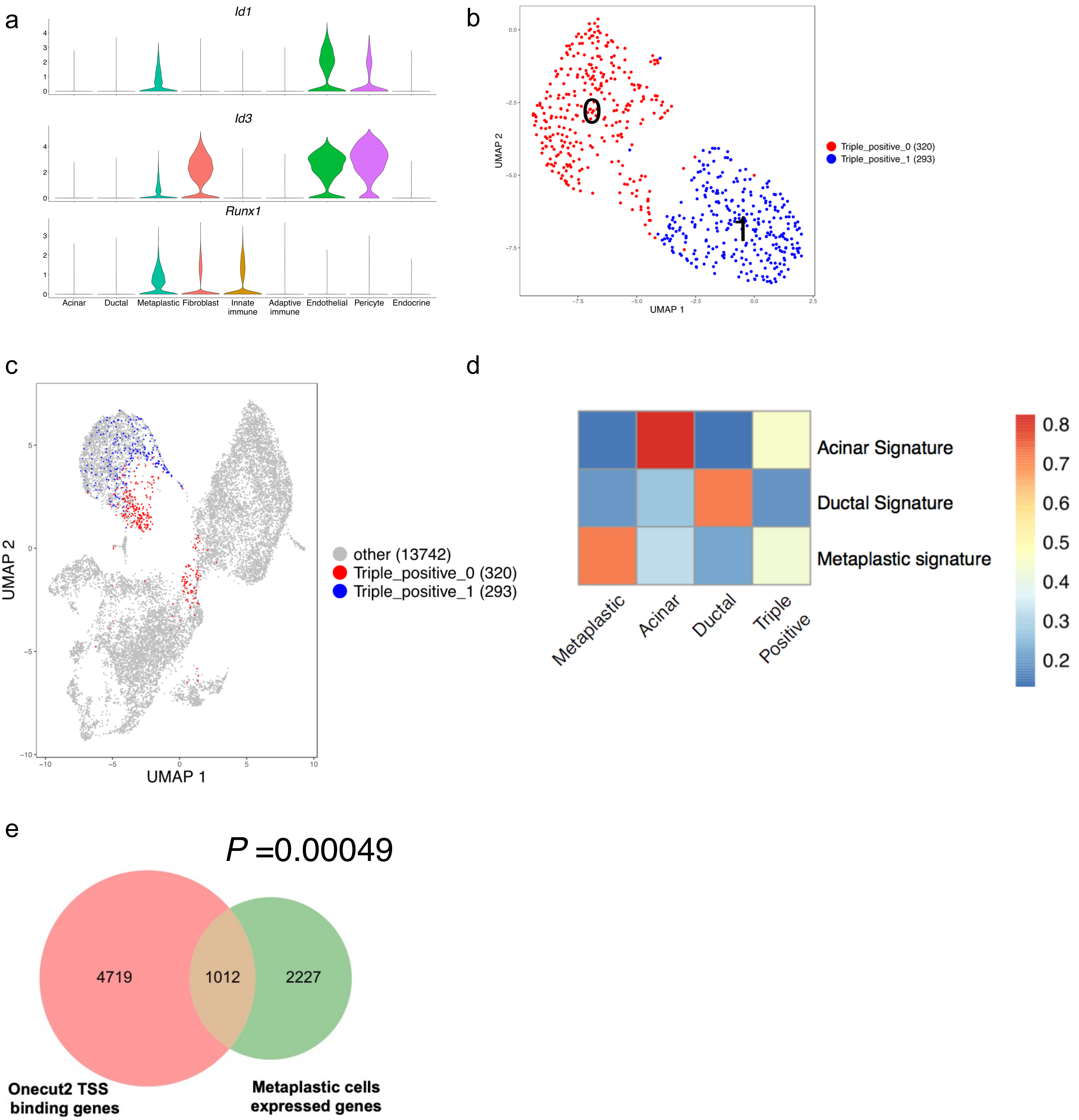

Name	p-value	Overlap	
Integrin Signaling	1.41E-15	19.2 %	41/213
ERK/MAPK Signaling	2.07E-13	18.7 %	36/193
EIF2 Signaling	9.80E-13	17.0 %	38/224
Germ Cell-Sertoli Cell Junction Signaling	4.09E-12	18.7 %	32/171
Molecular Mechanisms of Cancer	5.21E-12	13.0 %	51/391

Supplementary Fig. 3: Early metaplastic cells show an intermediate expression program between acinar and late metaplastic cells.

(a) Violin plot showing the expression level of *Id1*, *Id3* and *Runx1* in different cell types (x axis) in the mouse single-cell data.

(b) UMAP that includes all the cells that express *Cpa1*, *Krt19* and *tdTomato* ("triple positive").

(c) The localization of the "triple positive" cells shown in b in the UMAP that includes all the acinar ductal and metaplastic cells (Fig. 2a). Cells from cluster 1 are distributed in the acinar clusters while cells in cluster 0 are localized in two regions.

(d) Heat map showing the expression level of acinar, ductal and metaplastic signatures in different sub-populations of cells.

(e) Venn diagram showing the overlap between metaplastic versus acinar differentially-expressed genes and adjacent genes to Onecut2 binding location. P-value was calculated using a hypergeometric test. Annotations of overlapping genes (expressed and bound by Onecut2) were assign using IPA software, P-value (hypergeometric test) and number of (overlapping genes)/(total genes in the pathway) are shown.

Supplementary Fig. 4

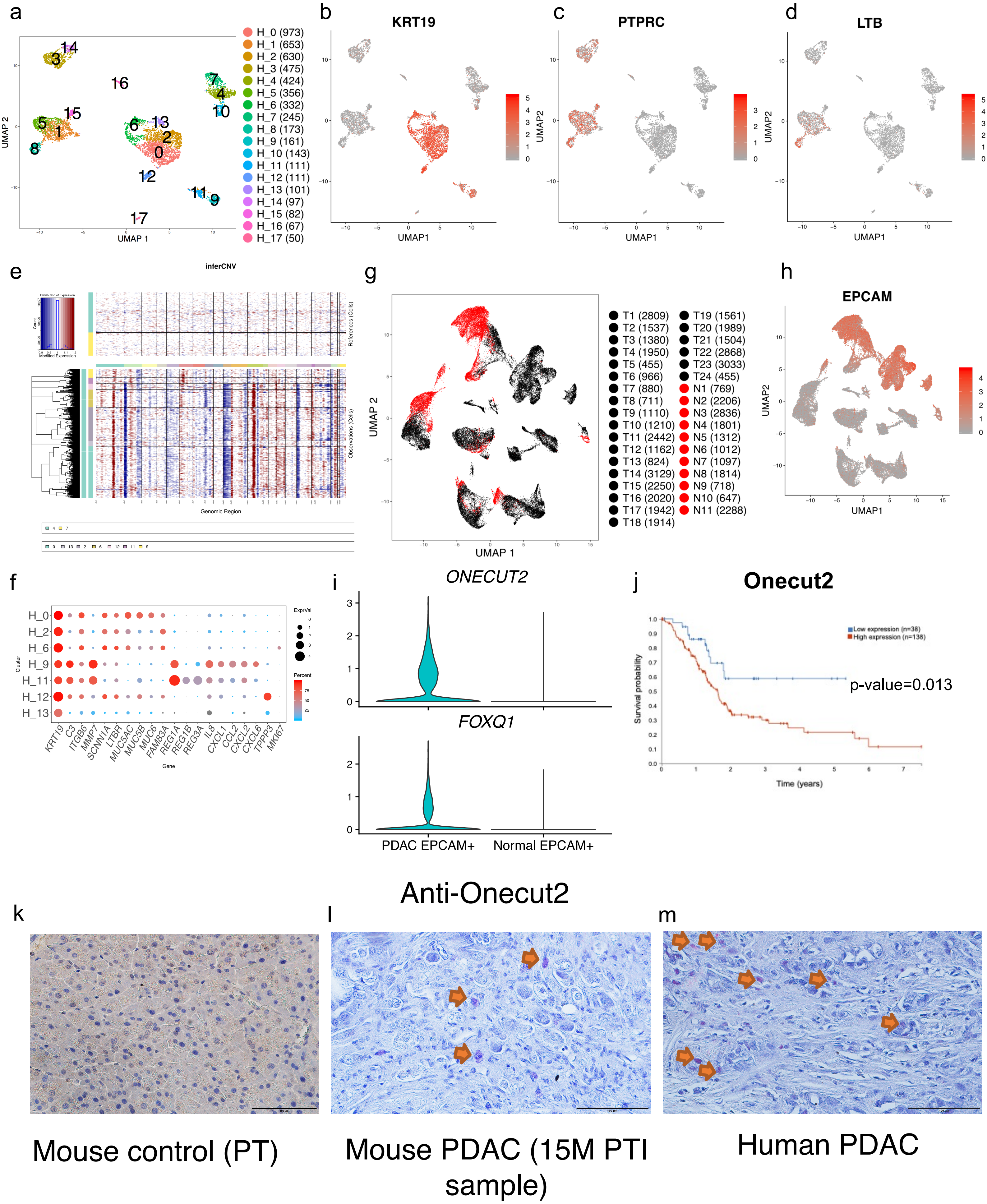

Supplementary Fig. 4: Single cell RNA-seq of human PDAC samples

(a) UMAP chart showing clusters of Human PDAC single cell analysis that was generated in this study. Clusters names and number of cells are shown.

(b-d) UMAP showing the expression level of selected genes. High expression level - red, low expression level - gray. Scale is on the right and relevant gene name on the top of each chart.

(e) Chromosome number variation analysis of human sample. On the X axis, chromosome numbers, on the Y axis *KRT19*-positive clusters numbers. On the top, two *KRT19*-negative clusters used as controls. Copy number score is indicated on the left.

(f) Dot plot chart showing the expression level of selected genes in human *KRT19*-positive clusters. The average expression level is shown by the size of the dot and the percentage of cells that express the relevant gene is indicated by the color.

(g) UMAP showing clusters of Human PDAC using data generated by *Peng et al* [24]. PDAC patients' cells are colored in black, cells that were recovered from healthy donors are colored in red.

(h) UMAP showing the expression level of EPCAM in the data that is presented in panel (g).

(i) Violin plot showing the expression level of *ONCUT2* and *FOXQ1* in Epcam-positive cells recovered from PDAC and healthy donors.

(j) Kaplan-Meier patient survival curve based on data obtained from the TCGA Pancreatic Adenocarcinoma (PAAD) dataset. The association between the level of *ONECUT2* and patient survival is shown. P –value was derived using the Log-rank test, test-statistic is chi-squared.

(k-m) Immunostaining with anti-Onecut2, (k) section from control mice, (l) section from 15 months PTI tumor bearing PRT mice, (m) section from human PDAC sample. Scale bars 100 μm.

Supplementary Fig. 5

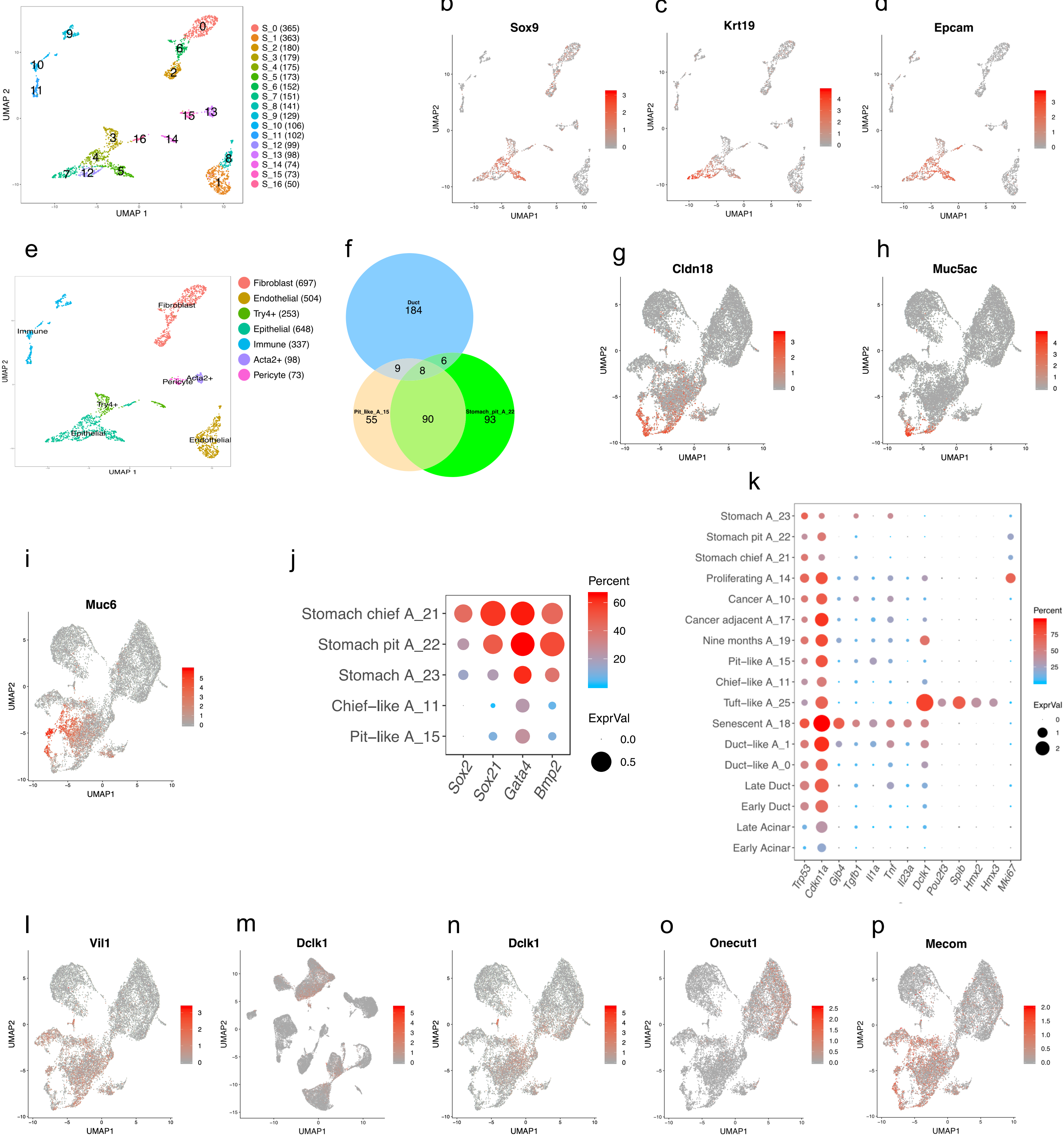

Supplementary Fig. 5: The expression of different genes in stomach and metaplastic cells

(a) UMAP showing a single cell RNA-seq experiment of stomach tissue from wild type mice. The number of each cluster is indicated and the numbers of cells in each cluster are shown on the right.

(b-d) UMAPs showing the expression level of representative markers for each cell type. (b) Sox9, (c) Krt19, (d) Epcam. High expression level - red, low expression level - grey. Scale is on the right and relevant gene names are at the top of each chart.

(e) Stomach single cell data colored according to cells identities.

(f) Scaled Venn diagram showing overlapping DE genes between clusters: A_15, A_22 and ductal cells. Numbers of genes are indicated.

(g) UMAP of acinar ductal and metaplastic cells, colored based on the expression of *Cldn18*. Stomach cells clusters and cluster A_15 shows high expression.

(h) UMAP of acinar ductal and metaplastic cells, colored based on the expression of *Muc5ac*. Stomach cells clusters and cluster A_15 shows high expression.

(i) UMAP of acinar ductal and metaplastic cells, colored based on the expression of *Muc6*. Stomach cells clusters and cluster A_11 show high expression.

(j) Dot plot showing the expression level of set of genes that are expressed in stomach cells. Clusters that include stomach cells and metaplastic stomach-like cells are shown on the Y axis. The average expression level is shown by the size of the dots and the percentage of cells is indicated by the color.

(k) Dot plot showing the expression of selected sets of genes in metaplastic cells, ductal cells and acinar cells as shown on the Y axis. The average expression level is shown by the size of the dots and the percentage of cells is indicated by the color. *Cdkn1a* encodes P21 and *Trp53* encodes P53.

(l-p) UMAP of acinar ductal and metaplastic cells showing the expression level of *Vil1*.

(m) UMAP of all cell types (based on Fig. 1i), colored based on the expression of *Dclk1*.

(n) UMAP of acinar ductal and metaplastic cells showing the expression level of *Dclk1*.

(o) UMAP of acinar ductal and metaplastic cells showing the expression level of *Onecut1*.

(p) UMAP of acinar ductal and metaplastic cells showing the expression level of *Mecom*.

Supplementary Fig. 6

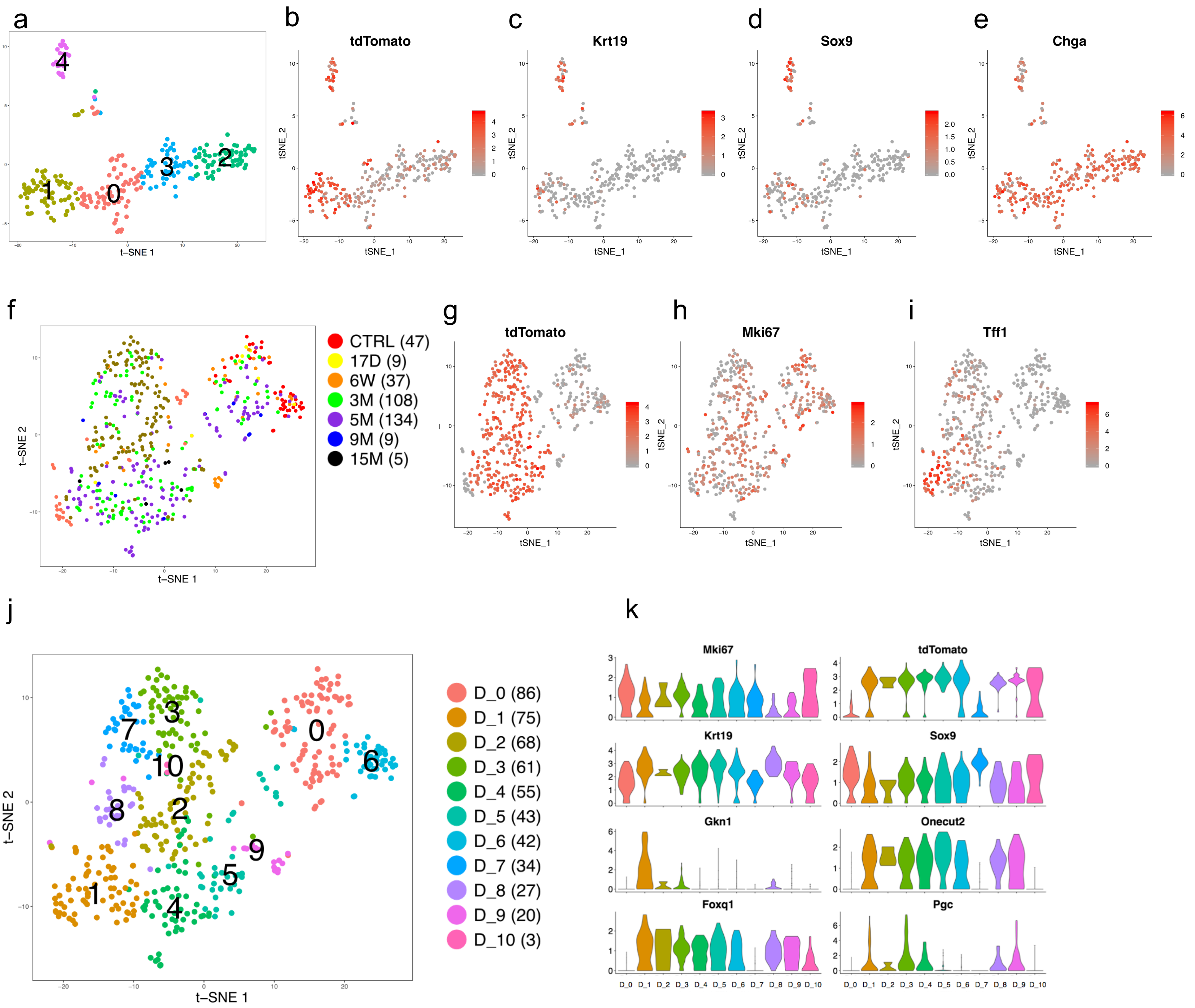

Supplementary Fig. 6: Reanalysis of neuroendocrine cells and metaplastic acinar and ductal cycling cells

(a) Reanalysis of neuroendocrine cells (cluster All_18 in supplementary Fig. 1i).

(b-e) UMAP showing the expression level of selected genes including ductal metaplastic and neuroendocrine markers. High expression level - red, low expression level - grey. (b) *tdTomato*, (c) *Krt19*, (d) *Sox9*, (e) *Chga*.

(f) Reanalysis of cells in cluster A_14 that includes cycling cells. Each time point PTI is colored differently and number of cells is indicated.

(g-i) UMAPs showing the expression level of selected genes in the UMAP presented in (f). High expression level - red, low expression level - grey. (g) *tdTomato*, (h) *Mki67*, (i) *Tff1*.

(j) UMAP presented in (f) colored based on cluster classification. The number of cells in each cluster is shown on the right. Clusters D_0 and D_6 include proliferating ductal cells.

(k) Violin plot representing the expression level of relevant set of markers of metaplastic cells in cycling cells. Clusters are on the X axis. Expression levels are on the Y axis.

Supplementary Fig. 7

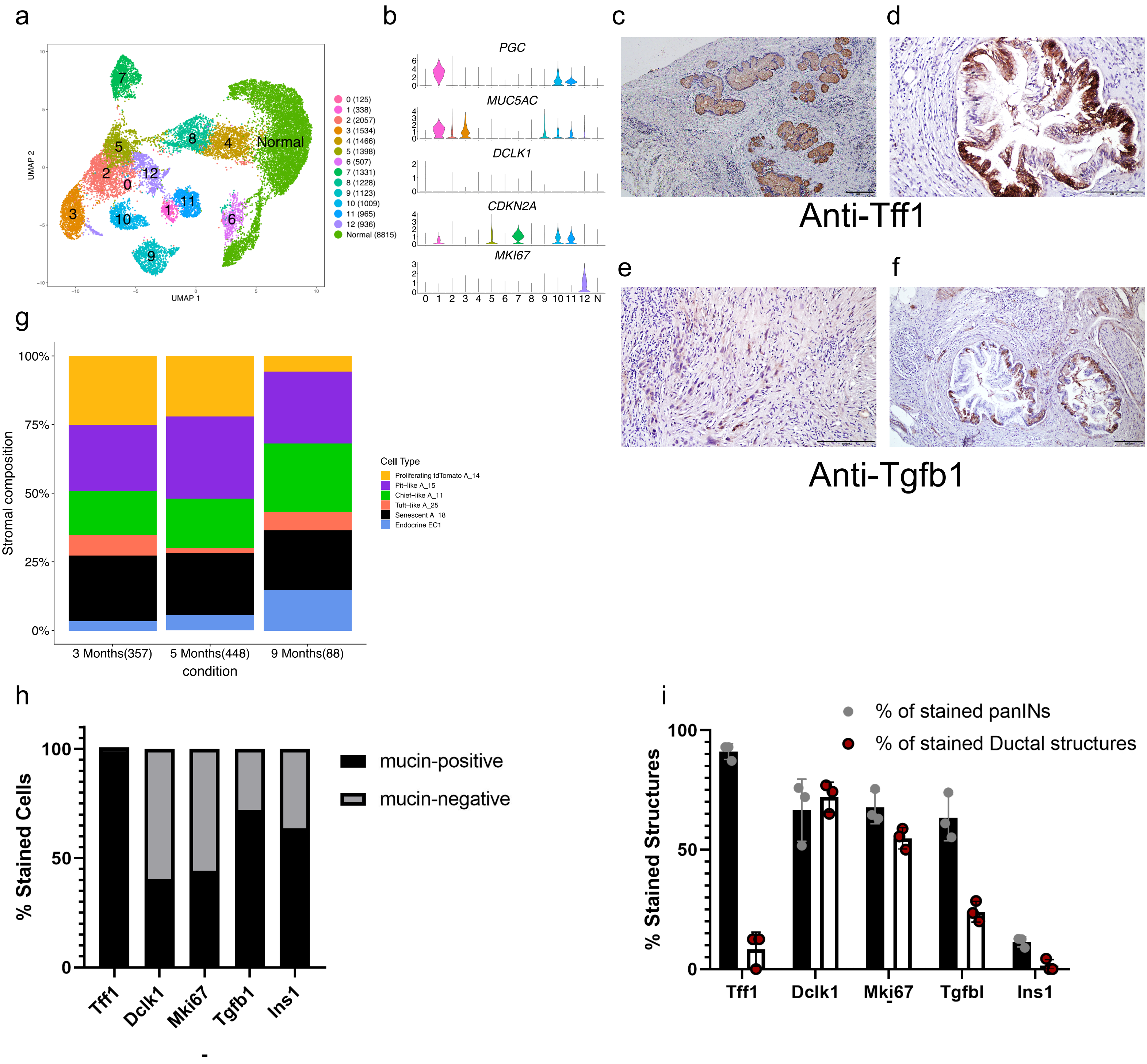

Supplementary Fig. 7: Expression of metaplastic cells markers in human PDAC samples and the relative distribution of metaplastic cell types in mice.

(a) Analysis of Krt19 positive clusters from human PDAC generated by *Peng et* al [24]. "Normal" mark cells that originate from the control, non-PDAC samples. Cluster numbers and cell numbers are indicated on the right.

(b) Violin plot showing the expression of metaplastic markers in the different clusters in (a).

(c-d) Immunohistochemistry of human PDAC sample using anti-Tff1 antibody, a marker of metaplastic pit-like cells. Scale bars 200 μm.

(e-f) Immunohistochemistry of human PDAC sample using anti-Tgfb antibody. Scale bars 200 μm.

(g) Bar plot showing the relative number of metaplastic cell-types based on the mice single cell data presented in this study. Late time points PTI are included and shown on the X axis.

(h) Graph showing the relative metaplastic cell-types markers in cells that are large and contain mucins and cells that do not contain mucins. Data collected from slices of three different mice for each antibody (n=3), and is based on counting of 39,241 cells, see Supplementary Data 9. The bars represent the mean values.

(i) Graph showing the percentage of PanINs or ductal structures that contain at least one cell from the indicated metaplastic cell-types. Data collected from slices of three different mice for each antibody (n=3), and is based on counting of 837 lesions, see Supplementary Data 9. The bars represent the mean values and +/- SD .

Supplementary Fig. 8

a

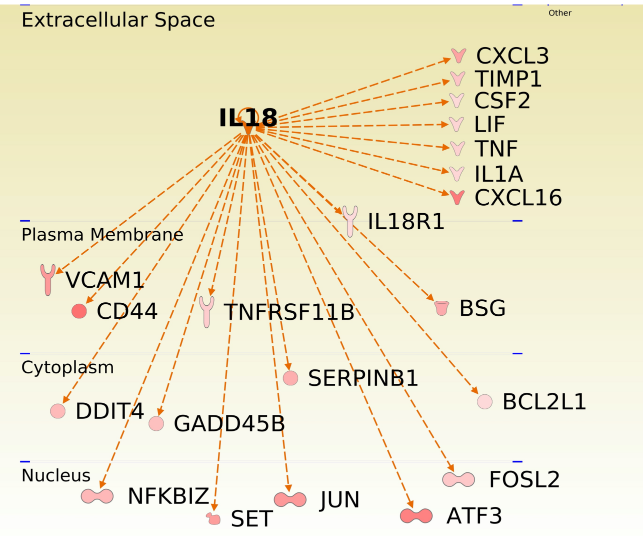

P-value = 10E−03

b

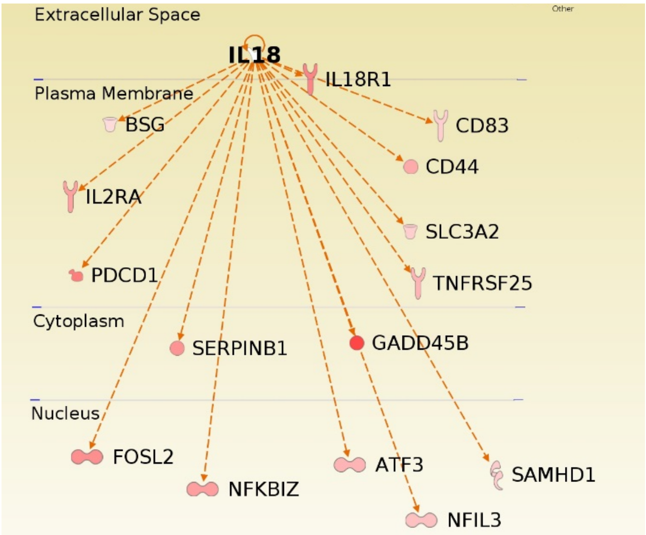

P-value = 2.74E−10

c Anti- CD3

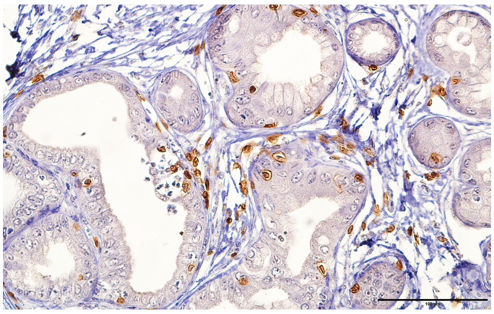

d Anti- CD3

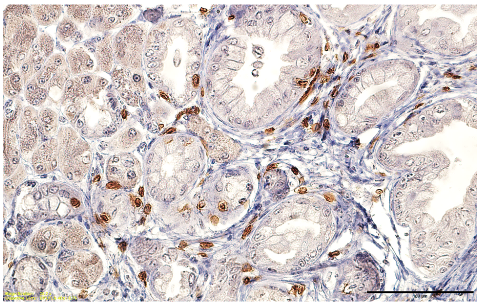

e Anti-Muc5ac Anti- CD3

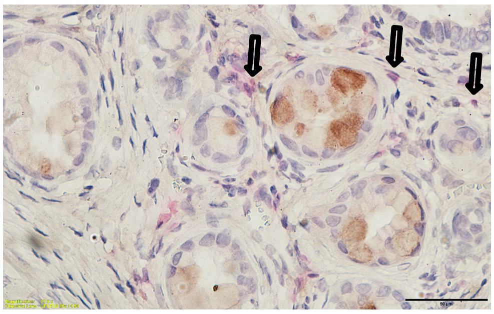

f Anti-Muc5ac Anti- CD3

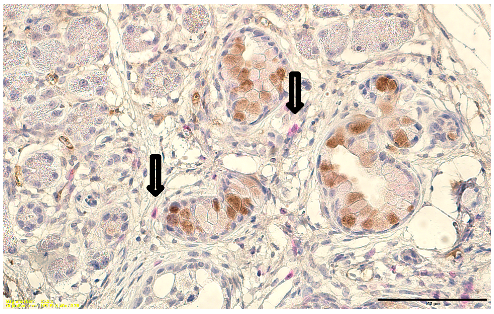

g Anti-Muc5ac Anti- Ly6g

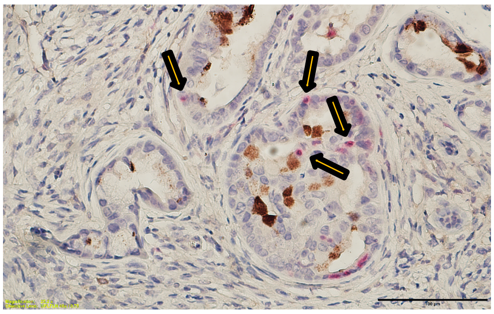

h Anti-Muc5ac Anti- Ly6g

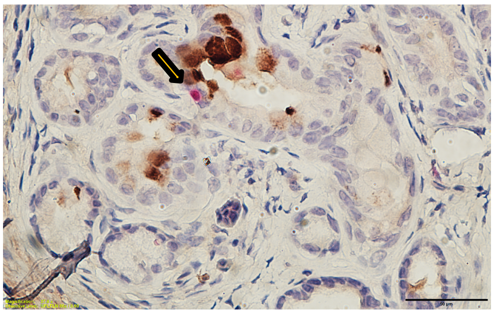

Supplementary Fig. 8: Cell-cell signaling and relative localization.

(a-b) The figure shows genes that respond to Il18 signaling based on the IPA database. (a) Overlap with senescent expressed genes (cluster A_18). (b) Overlap with CD4 T cells expressed genes. P-value based on hypergeometric test is indicated at the bottom of each panel.

(c-d) Staining of section taken from the pancreas of a five month PTI PRT mouse. Staining using anti-CD3 showing association between T cells and PaniNs. Scale bars 100 µm.

(e-f) Immunostaining of histological sections taken from the pancreas of a five month PTI PRT mouse, with anti-CD3 (red) and anti-Muc5ac (brown) antibodies. Arrows mark representative cells that stained positive with anti-CD3. (e) Scale bar 50 µm. (f) Scale bar 100µm.

(g-h) Immunostaining of histological sections taken from the pancreas of a five month PTI PRT mouse, with anti-LY6G (red) and anti-Muc5ac (brown) antibodies. Arrows mark representative cells that stained positive with anti-LY6G. (g) Scale bar 100 µm. (h) Scale bar 50 µm.

Supplementary Fig. 9

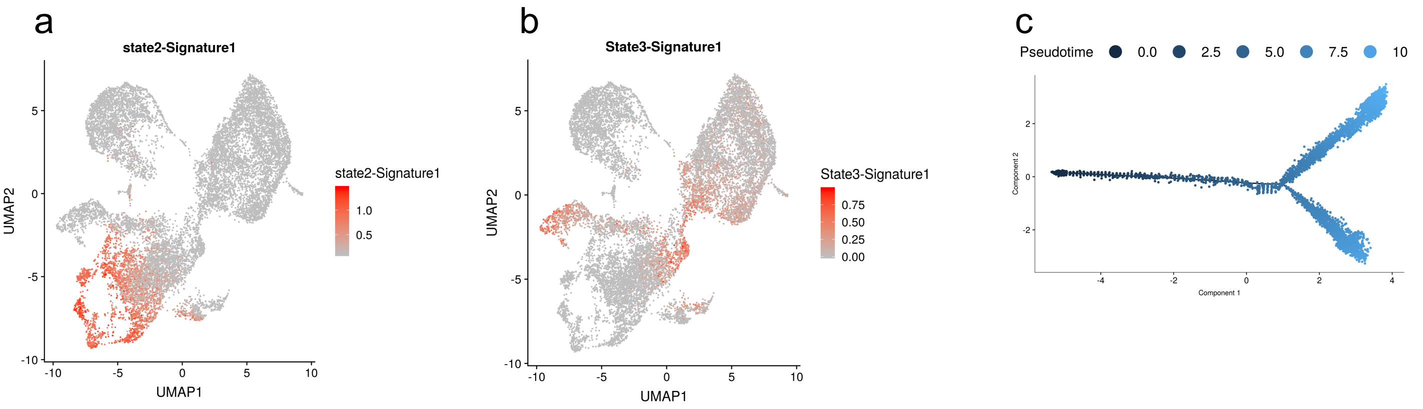

Supplementary Fig. 9: Distinct signature of metaplastic cells states

(a-b) UMAP showing the signature score (in red) of state 2 and state 3 (see Fig. 6).

(c) Trajectory chart showing pseudotime score for acinar, metaplastic and tumor cells.

Supplementary Fig. 10

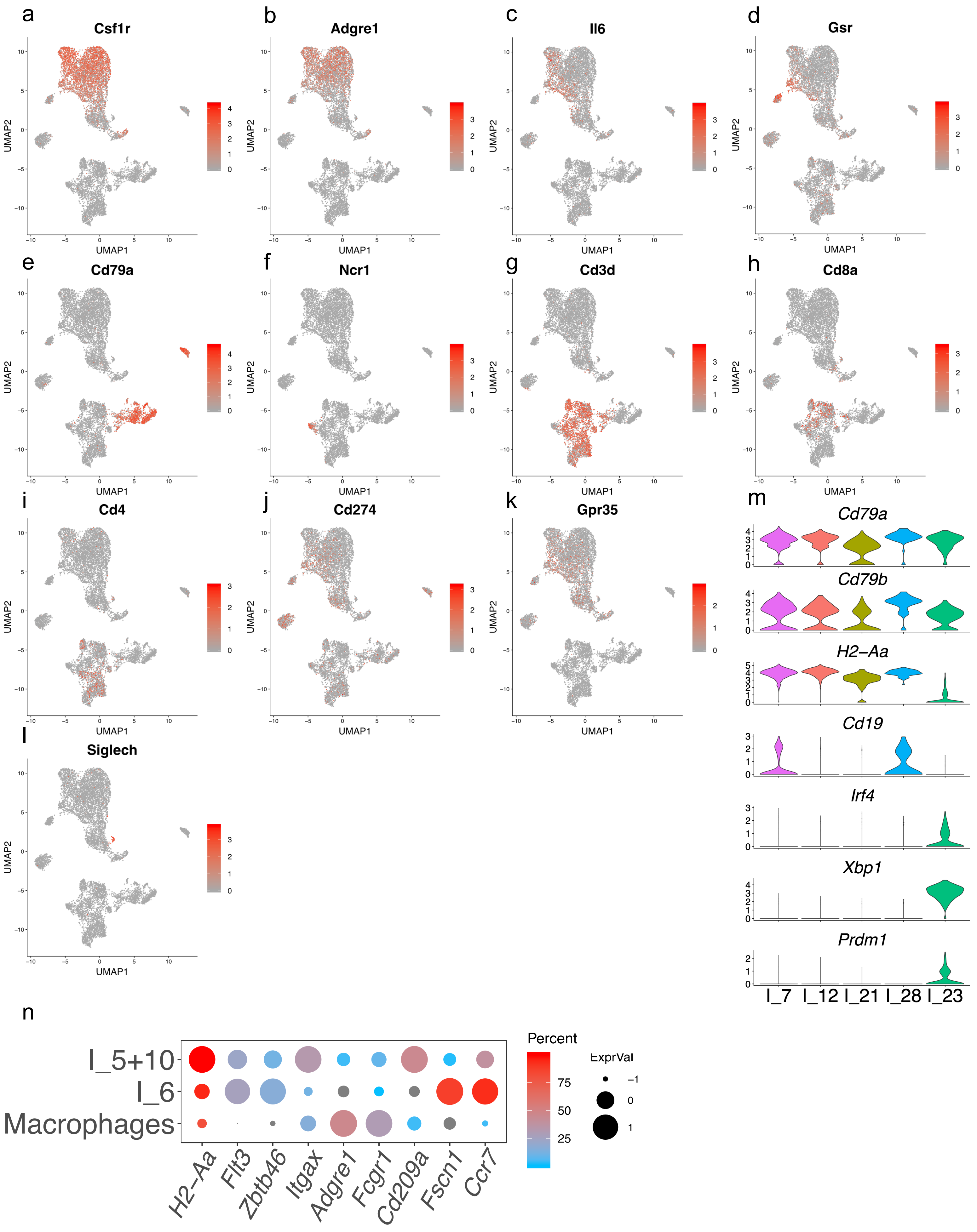

Supplementary Fig. 10: Immune cells change along the development of PanINs and cancer

(a-l) UMAP showing the expression level of selected genes that express in immune cells, macrophages (a-c, k), neutrophils (d), B cells (e), NK cells (f), T cells (g-i), pDCs (l).

(m) Violin plot representing the expression level of selected genes that are expressed in B cells. X axis - B cell clusters. Y axis - normalized expression.

(n) Dot plot showing the expression of set of immune related genes in macrophages and DCs clusters. The average expression level is scaled and shown by the size of the dots and the percentage of cells is indicated by the color.

Supplementary Fig. 11

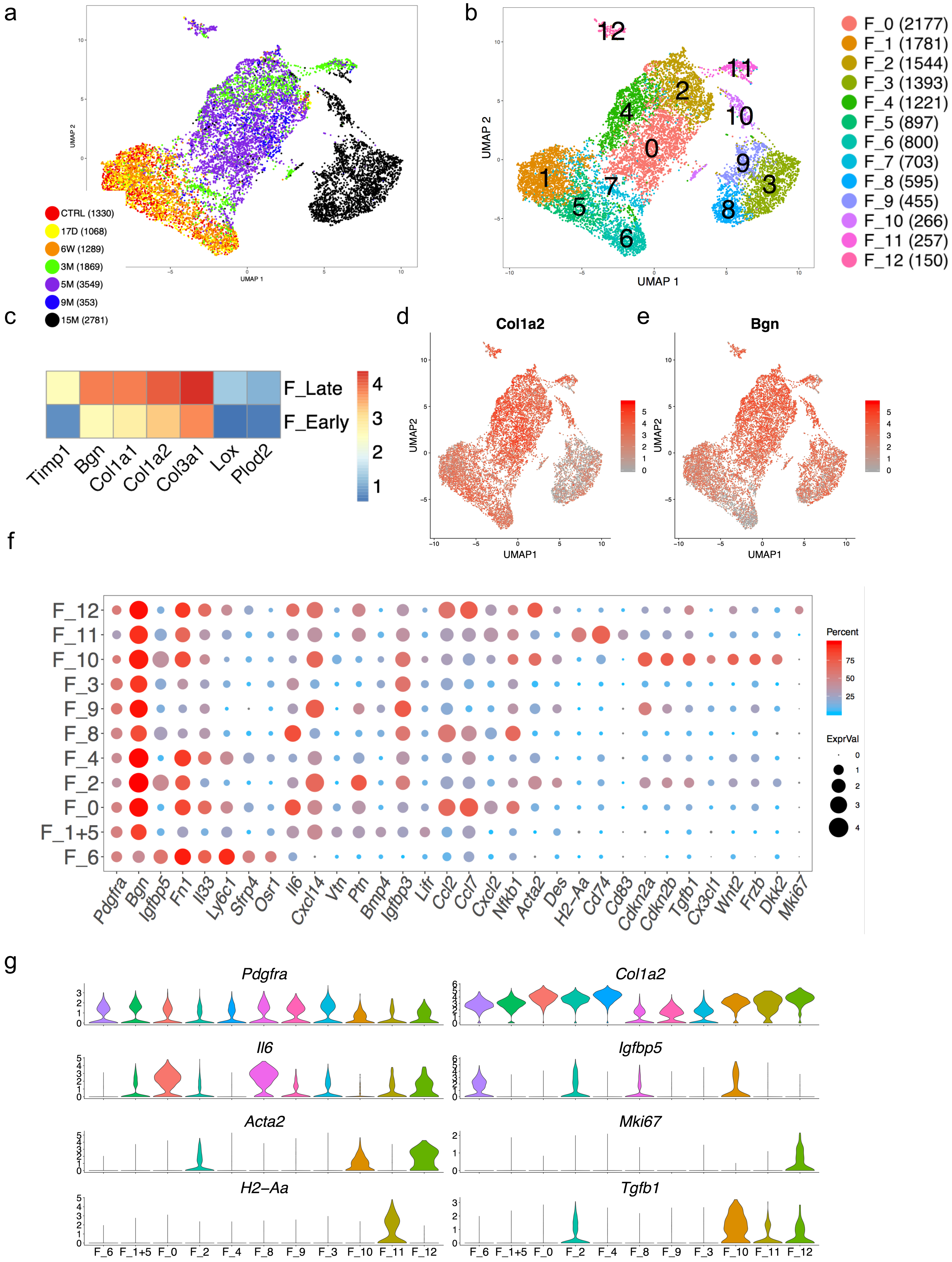

Supplementary Fig. 11: Fibroblast heterogeneity PTI injection

(a) Reanalysis of fibroblasts (Col1a1-positive clusters). Time points post tamoxifen injection and cell numbers are presented on the left.

(b) The same UMAP as in (a) the number of each cluster are shown. Cluster numbers and cell numbers in each cluster are presented on the right.

(c) Differentially expressed genes between early time points PTI and late time points PTI in fibroblast cells. Expression scale is indicated on the right and gene name on the bottom.

(d-e) UMAP showing the expression level of *Col1a2* and *Bgn*, representative genes that contribute to the desmoplastic phenotype.

(f) Dot plot showing the expression of set of genes (X axis) in different clusters of cells (Y axis). The average expression level is shown by the size of the dot and the percentage of cells that express the relevant gene is indicated by the color.

(g) Violin plot showing the expression level of selected genes in different fibroblasts clusters. Y axis - normalized expression.
