# Supplementary material for: Single-cell transcriptomes of pancreatic preinvasive lesions and cancer reveal acinar metaplastic cells’ heterogeneity
Source: Nat Commun. 2020 Sep 9;11:4516. doi: 10.1038/s41467-020-18207-z (PMC7481797; doi:10.1038/s41467-020-18207-z)
Supplement: Supplementary file 3 — Reporting Summary [file 41467_2020_18207_MOESM3_ESM.pdf]

nature research

Corresponding author(s): Oren Parnas, Gideon Zamir

Last updated by author(s): Jul 29, 2020

Reporting Summary

Nature Research wishes to improve the reproducibility of the work that we publish. This form provides structure for consistency and transparency in reporting. For further information on Nature Research policies, see our Editorial Policies and the Editorial Policy Checklist.

Statistics

For all statistical analyses, confirm that the following items are present in the figure legend, table legend, main text, or Methods section.

n/a	Confirmed	
☐	☒	The exact sample size (*n*) for each experimental group/condition, given as a discrete number and unit of measurement
☐	☒	A statement on whether measurements were taken from distinct samples or whether the same sample was measured repeatedly
☐	☒	The statistical test(s) used AND whether they are one- or two-sided<br>*Only common tests should be described solely by name; describe more complex techniques in the Methods section.*
☐	☒	A description of all covariates tested
☐	☒	A description of any assumptions or corrections, such as tests of normality and adjustment for multiple comparisons
☐	☒	A full description of the statistical parameters including central tendency (e.g. means) or other basic estimates (e.g. regression coefficient) AND variation (e.g. standard deviation) or associated estimates of uncertainty (e.g. confidence intervals)
☐	☒	For null hypothesis testing, the test statistic (e.g. *F*, *t*, *r*) with confidence intervals, effect sizes, degrees of freedom and *P* value noted<br>*Give P values as exact values whenever suitable.*
☒	☐	For Bayesian analysis, information on the choice of priors and Markov chain Monte Carlo settings
☐	☒	For hierarchical and complex designs, identification of the appropriate level for tests and full reporting of outcomes
☐	☒	Estimates of effect sizes (e.g. Cohen's *d*, Pearson's *r*), indicating how they were calculated

Our web collection on statistics for biologists contains articles on many of the points above.

Software and code

Policy information about availability of computer code

Data collection	bcl2fastq (v1.8.3) , Cell Ranger pipeline (v3.0.2 10x Genomics)
Data analysis	We used the following software/Packages:<br>monocle R package (v2.9.0)<br>inferCNV R package(v1.0.4)<br>ChIPseeker R package(v3.11)<br>eulerr R package (v6.1.0)<br>pheatmap R package (v1.0.12)<br>ggplot2 R package (v3.3.0)<br>Seurat R package (v2.3.4)<br>scipy python package (v1.4.1)<br>GraphPad Prism version v8.0.0 for Windows<br>IPA Qiagen software (https://digitalinsights.qiagen.com/products-overview/discovery-insights-portfolio/analysis-and-visualization/qiagen-ipa/)

For manuscripts utilizing custom algorithms or software that are central to the research but not yet described in published literature, software must be made available to editors and reviewers. We strongly encourage code deposition in a community repository (e.g. GitHub). See the Nature Research guidelines for submitting code & software for further information.

Data

Policy information about availability of data

All manuscripts must include a data availability statement. This statement should provide the following information, where applicable:

- Accession codes, unique identifiers, or web links for publicly available datasets
- A list of figures that have associated raw data
- A description of any restrictions on data availability

GEO Series accession number
GSE141017 (https://www.ncbi.nlm.nih.gov/geo/query/acc.cgi?acc=GSE141017)

Field-specific reporting

Please select the one below that is the best fit for your research. If you are not sure, read the appropriate sections before making your selection.

☒ Life sciences ☐ Behavioural & social sciences ☐ Ecological, evolutionary & environmental sciences

For a reference copy of the document with all sections, see nature.com/documents/nr-reporting-summary-flat.pdf

Life sciences study design

All studies must disclose on these points even when the disclosure is negative.

Sample size	The time-points of single-cell experiments were chosen based on the histological changes in the tissue. Duplicates of three months and five months post tamoxifen injection time point show similar results as presented in Supplementary figure 1 B-C (i.e cells from the different replicates cluster together). In addition, as we state in the first paragraph of the Results section, cells that were taken from the early time points in which there are very few lesions(17d, 6w, and control), cluster together. It strongly suggests that there are no batch effects. The same is true for the late time points in which the tissue contains many lesions (three months, five months, and nine months), the pathology is similar and the cells cluster according to the cell type and not according to the time point. Based on these observations we conclude that additional repeats will not change any of the conclusions. In addition, this mice model is well synchronized because it develops very slowly, different animals post tamoxifen injection show very similar lesion and pathology.<br>The number of single cells that we included in the manuscript is very high around 50000 cells, and support the conclusion that our data is reproducible.
Data exclusions	No data were excluded from the analysis.
Replication	All the single-cell experiments that technically succeeded (live cells) were successful and biological replicate cluster together. Immunostaining of slides from different mice gave similar results and replicates of histological sections from the same time points were similar.
Randomization	We randomly chose mice, with the required genotype, to single-cell experiments. Samples for histology were recovered from the same mice. Humans with positive PDAC biopsy were chosen randomly for single-cell experiments and histology.
Blinding	For each sample of single-cell RNA-seq data, we apply the same pipeline without making any modification that relates to the origin of the sample or the timepoint. The consolidation of all the data from different time points was also done blindly.

Reporting for specific materials, systems and methods

We require information from authors about some types of materials, experimental systems and methods used in many studies. Here, indicate whether each material, system or method listed is relevant to your study. If you are not sure if a list item applies to your research, read the appropriate section before selecting a response.

Materials & experimental systems

n/a	Involved in the study
☐	☒ Antibodies
☒	☐ Eukaryotic cell lines
☒	☐ Palaeontology and archaeology
☐	☒ Animals and other organisms
☐	☒ Human research participants
☒	☐ Clinical data
☒	☐ Dual use research of concern

Methods

n/a	Involved in the study
☒	☐ ChIP-seq
☒	☐ Flow cytometry
☒	☐ MRI-based neuroimaging

Antibodies

Antibodies used	antibody catalog_number lot clone supplier name

Tff1 Ab190942 GR3251654-1 polyclonal abcam
CD3 MCA1477T 147806 CD3-12 biorad
CD3 ab21703 GR3304541-1 SP7 abcam
F4/80 MCA497RT 148452 CI:A3-1 biorad
Dclk1 Ab37994 GR313013-10 polyclonal abcam
Mki67 AB15580 GR3237546-1 polyclonal abcam
Muc5ac Ab3649 GR3213384-2 45M1 abcam
Gkn1 PA-47913 UA2703409A polyclonal thermofisher
Tgfb1 Ab92486 GR3273406-3 polyclonal abcam
Ins1 A0564 11146789 polyclonal "Dako"
Ly6g 551459 6160513 1A8 (RUO) BD Pharmingen
Ck19 Ab52625 GR3255534-1 EP1580Y abcam
Onecut2 Ab28466 GR3251021-14 polyclonal abcam

Validation

antibody website
Tff1 https://www.abcam.com/estrogen-inducible-protein-ps2-antibody-ab190942.html?productWallTab=ShowAll
CD3 https://www.bio-rad-antibodies.com/static/datasheets/mca14/human-cd3-antibody-cd3-12-mca1477t.pdf
CD3 https://www.abcam.com/cd3-antibody-sp7-prediluted-ab21703.html?productWallTab=ShowAll
F4/80 https://www.bio-rad-antibodies.com/static/datasheets/mca49/mouse-f4-80-antibody-cl-a3-1-mca497rt.pdf
Dclk1 https://www.abcam.com/dcamkl1-antibody-ab37994.html The antibody is not available anymore
Mki67 https://www.abcam.com/ki67-antibody-ab15580.html
Muc5ac https://www.abcam.com/mucin-5ac-antibody-45m1-ab3649.html?productWallTab=ShowAll
Gkn1 https://www.thermofisher.com/order/genome-database/dataSheetPdf?productType=antibody&productsubtype=antibody_primary&productId=PA5-47913&version=105
Tgfb1 https://www.abcam.com/tgf-beta-1-antibody-ab92486.html
Ins1 https://www.citeab.com/antibodies/3382917-a0564-insulin
Ly6g https://www.bdbiosciences.com/ds/pm/tds/551459.pdf
Ck19 https://www.abcam.com/cytokeratin-19-antibody-ep1580y-cytoskeleton-marker-ab52625.html?productWallTab=ShowAll
Onecut2 https://www.abcam.com/onecut2oc2-antibody-ab28466.html

Animals and other organisms

Policy information about studies involving animals; ARRIVE guidelines recommended for reporting animal research

Laboratory animals

The following strained from Jackson laboratory were crossed to create the mouse strain that we have used: JAX 007908, JAX 019378 and JAX 008179. 6 weeks female and male mice were induced for different durations as indicated in the paper. C57BL/6JOlaHsd was purchased from Envigo 9 weeks female (stomach single cell data).

Wild animals

No wild animals were used.

Field-collected samples

No field-collected samples

Ethics oversight

The joint ethics committee (Institutional Animal Care and Use Committee) of the Hebrew University (Jerusalem, Israel) and Hadassah Medical Center (Jerusalem, Israel) approved the study protocol for animal welfare. The Hebrew University is an Association for Assessment and Accreditation of Laboratory Animal Care International–accredited institute.

Note that full information on the approval of the study protocol must also be provided in the manuscript.

Human research participants

Policy information about studies involving human research participants

Population characteristics

Patients undergoing pancreatic resection for pancreatic adenocarcinoma. The only patient sample used for single cell RNA-seq in this study was collected from 80 years old man.

Recruitment

Recruitment was performed following informed consent. The study was approved by the Hadassah medical center IRB. .PDAC resection specimens were obtained from the Hebrew University-Hadassah Medical Center (Jerusalem, Israel). The studies were conducted in accordance with ethical guidelines (0327-17HMO Declaration of Helsinki). There was no bias in patient selection, the only requirement was biopsy that is positive to PDAC.

Ethics oversight

Human PDAC resection specimens were obtained from the Hebrew University-Hadassah Medical Center (Jerusalem, Israel). The studies were conducted in accordance with ethical guidelines (0327-17HMO Declaration of Helsinki).

Note that full information on the approval of the study protocol must also be provided in the manuscript.
