# Supplementary material for: Single-cell transcriptomes of pancreatic preinvasive lesions and cancer reveal acinar metaplastic cells’ heterogeneity
Source: Nat Commun. 2020 Sep 9;11:4516. doi: 10.1038/s41467-020-18207-z (PMC7481797; doi:10.1038/s41467-020-18207-z)
Supplement: Supplementary file 4 — Description of Additional Supplementary Files [file 41467_2020_18207_MOESM4_ESM.pdf]

Description of Additional Supplementary Files

Supplementary Data 1:
Differential expressed (DE) genes – all cell types.

Supplementary Data 2:
Differential expressed genes – between metaplastic cells and acinar cells.

Supplementary Data 3:
Differential expressed genes – between metaplastic cells and ductal cells.

Supplementary Data 4:
Differential expressed genes – acinar, ductal, metaplastic and stomach cells.

Supplementary Data 5:
Differential expressed genes – between acinar cells and early metaplastic cells (“triple positive”).

Supplementary Data 6:
Differential expressed genes – between early metaplastic cells (“triple positive”) and late metaplastic cells.

Supplementary Data 7:
Differential expressed genes – human PDAC sample.

Supplementary Data 8:
Differential expressed genes and overlap expressed genes between stomach pit cells and metaplastic pit-like.

Supplementary Data 9:
Quantities analysis of metaplastic cell-types and states.

Supplementary Data 10:
Differential expressed genes in immune and stromal cells
