# Supplementary material for: Single-cell transcriptomes of pancreatic preinvasive lesions and cancer reveal acinar metaplastic cells’ heterogeneity
Source: Nat Commun. 2020 Sep 9;11:4516. doi: 10.1038/s41467-020-18207-z (PMC7481797; doi:10.1038/s41467-020-18207-z)

Single cell transcriptomes of pancreatic pre-invasive lesions and cancer reveal acinar metaplastic cells’ heterogeneity

The data source file includes additional microscopy images from different mice and human patients and raw data of lesion counts that are shown in Supplementary Figure 1A. In the last part, we show the gating strategy for the tomato sorted cells.

Additional represented photos for Figure 1A

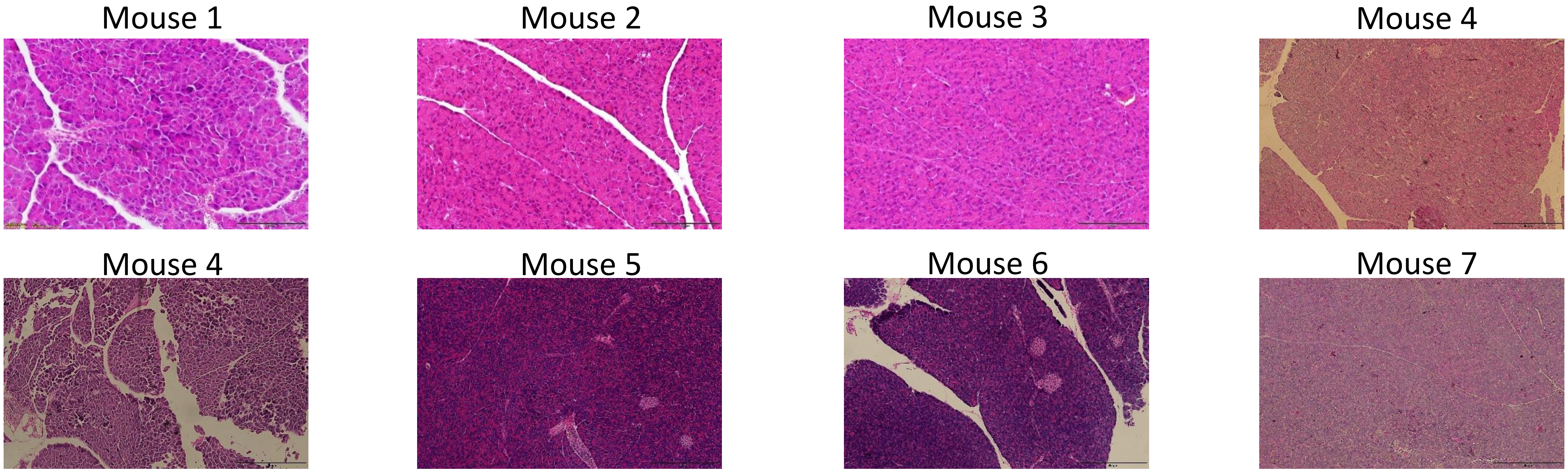

Additional represented photos for Figure 1B

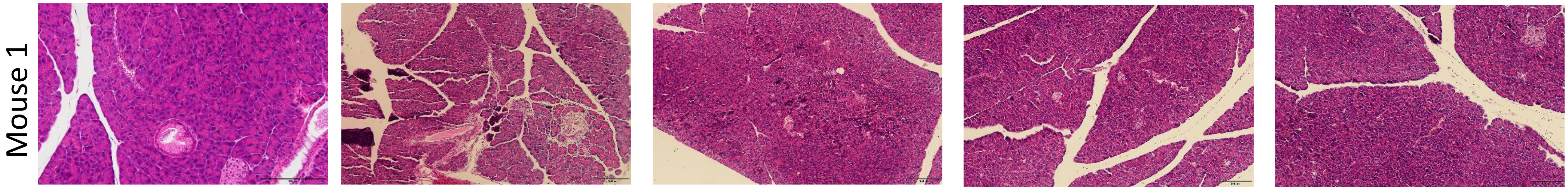

Additional represented photos for Figure 1C

Mouse 1

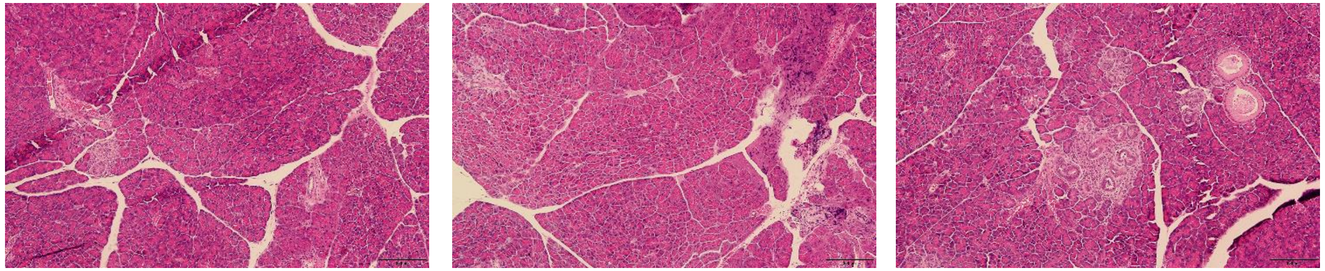

Mouse 2

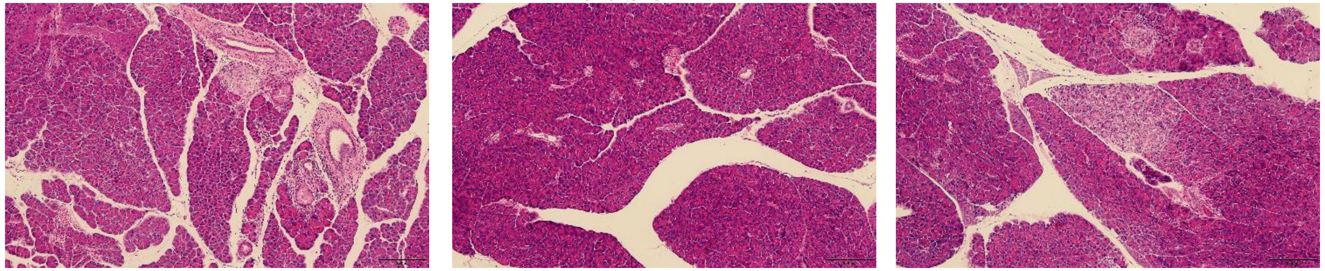

Additional represented photos for Figure 1D

Mouse 1

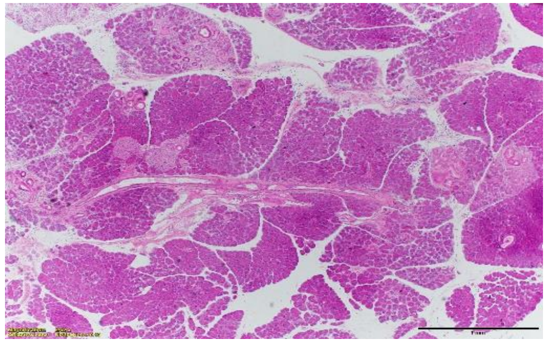

Mouse 2

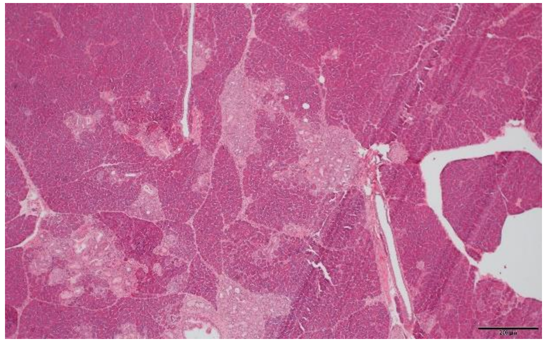

Mouse 3

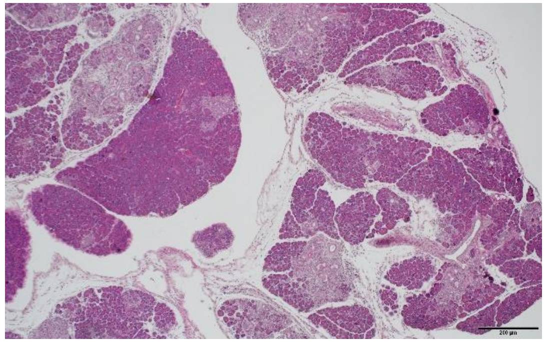

Mouse 4

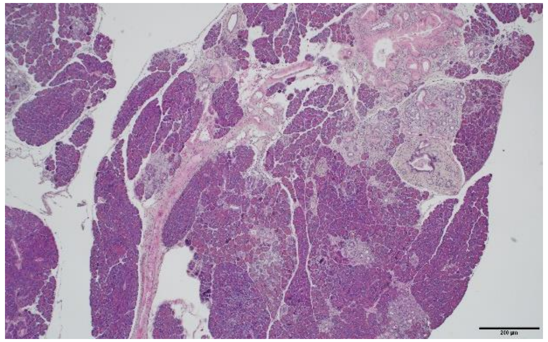

Additional represented photos for Figure 1E

Mouse 1

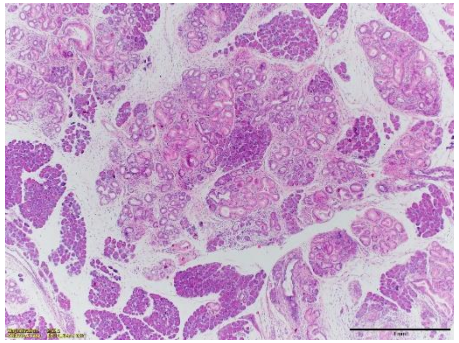

Mouse 2

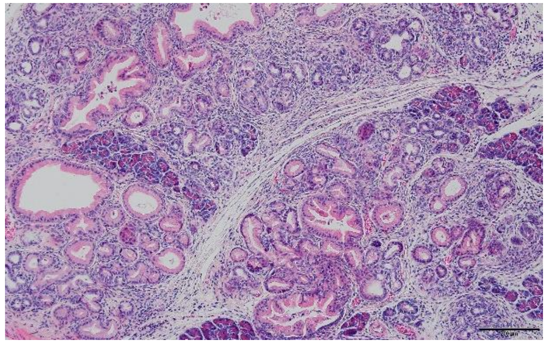

Mouse 3

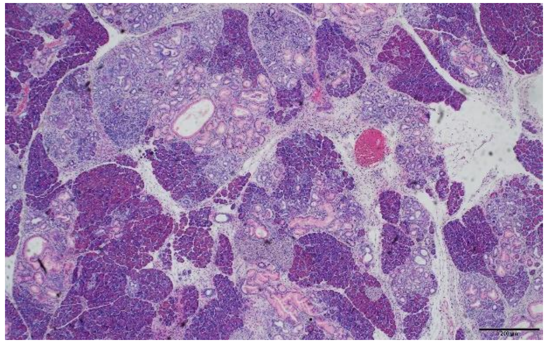

Mouse 4

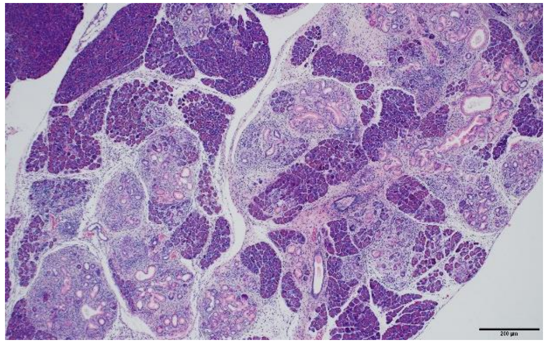

Mouse 5

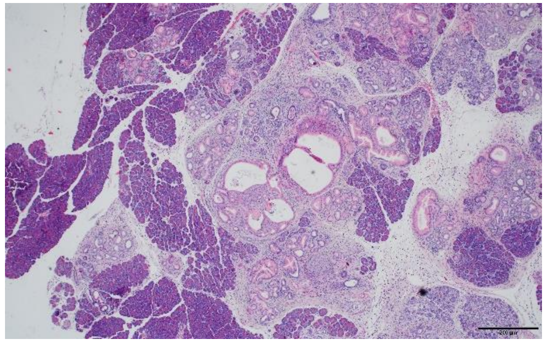

Additional represented photos for Figure 1F

Mouse 1

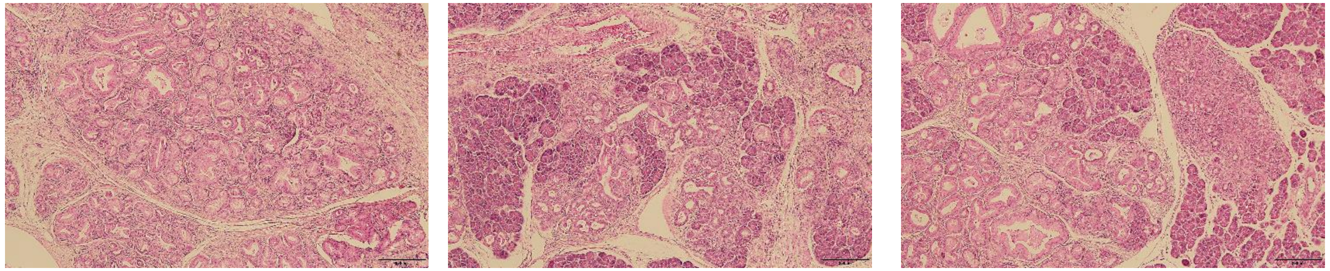

Additional represented photos for Figure 1G

Mouse 1

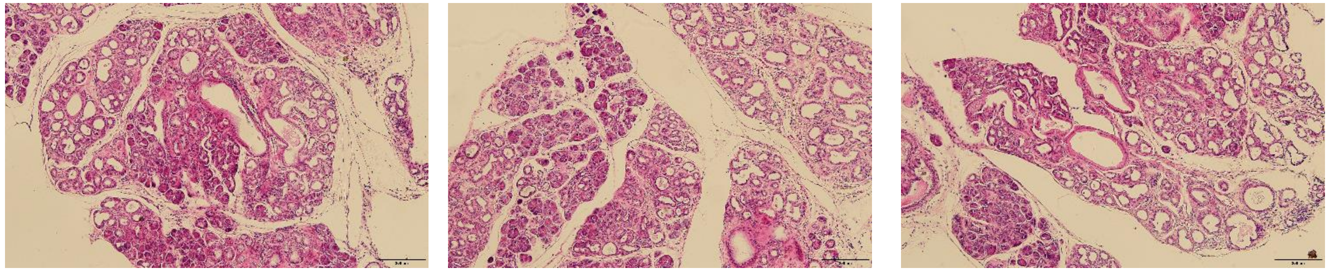

Additional represented photos for Figure 1H

Mouse 1

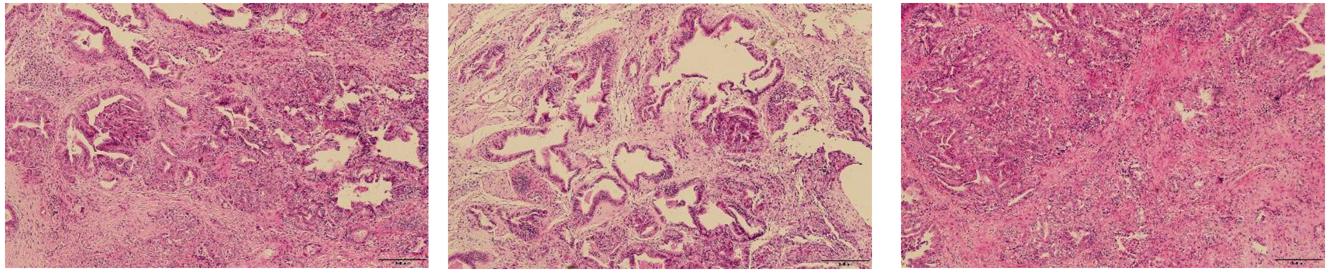

Additional represented photos for Figure 1J

Mouse 1 Mouse 2 Mouse 3

Anti-CD3

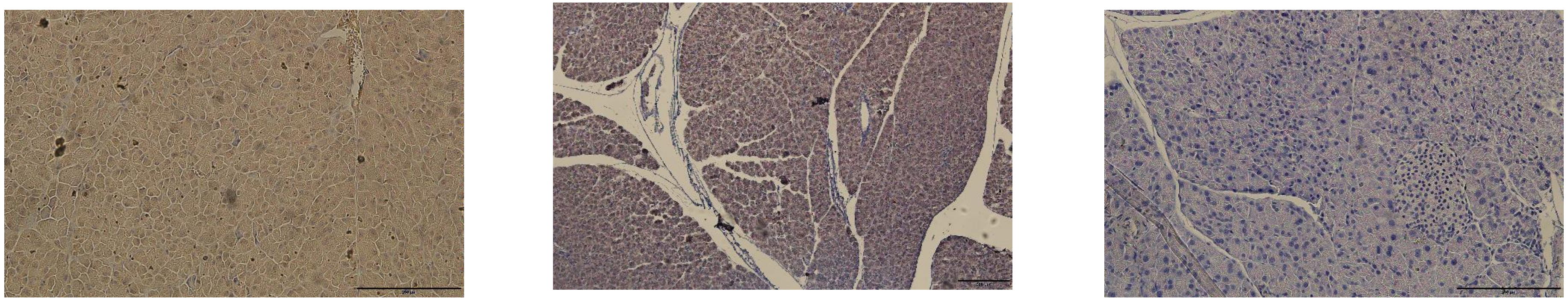

Additional represented photos for Figure 1K

Mouse 1 Mouse 2 Mouse 3

Anti-CD3

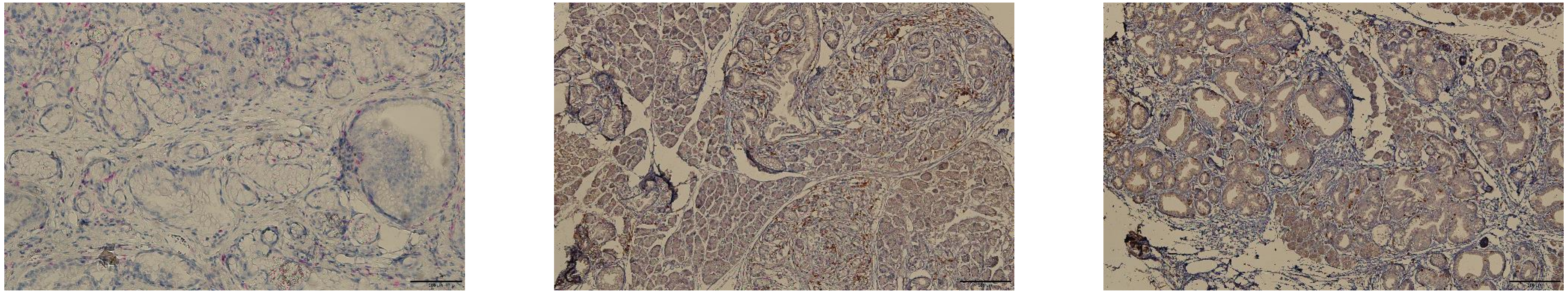

Additional represented photos for Figure 1L

Anti- F4/80

Mouse 1

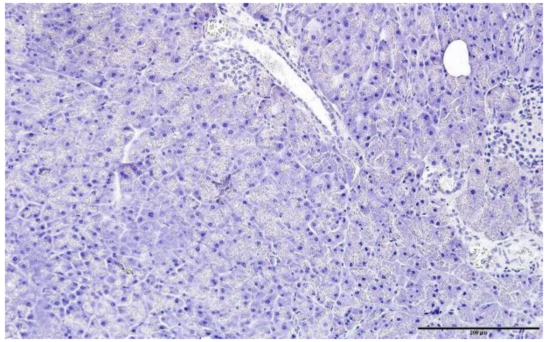

Mouse 2

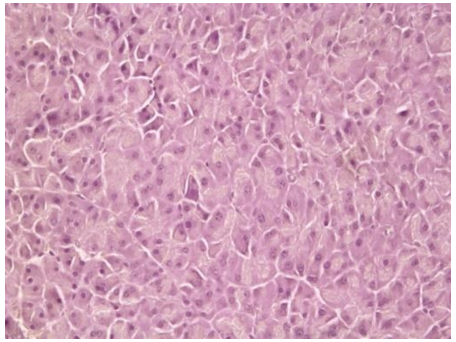

Additional represented photos for Figure 1M

Anti- F4/80

Mouse 1

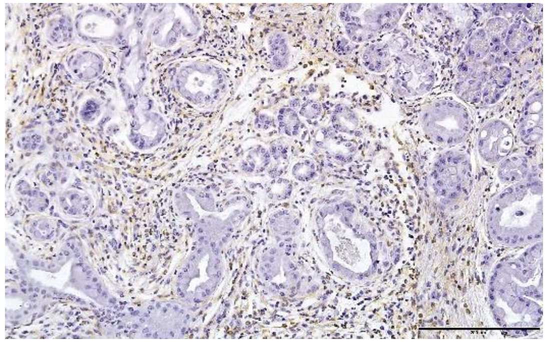

Mouse 2

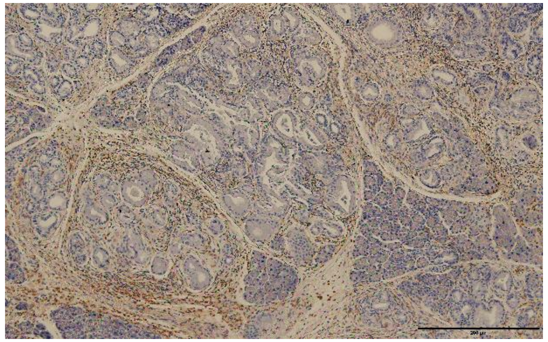

Mouse 3

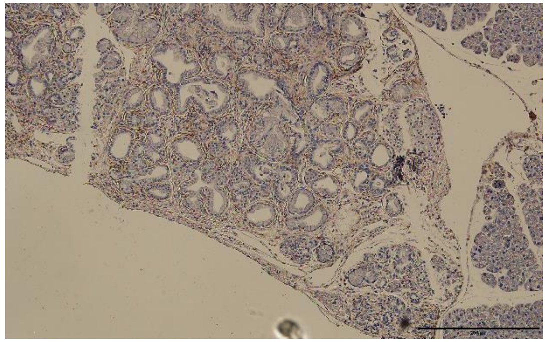

Additional represented photos for Figure 3F

Anti-GKN1

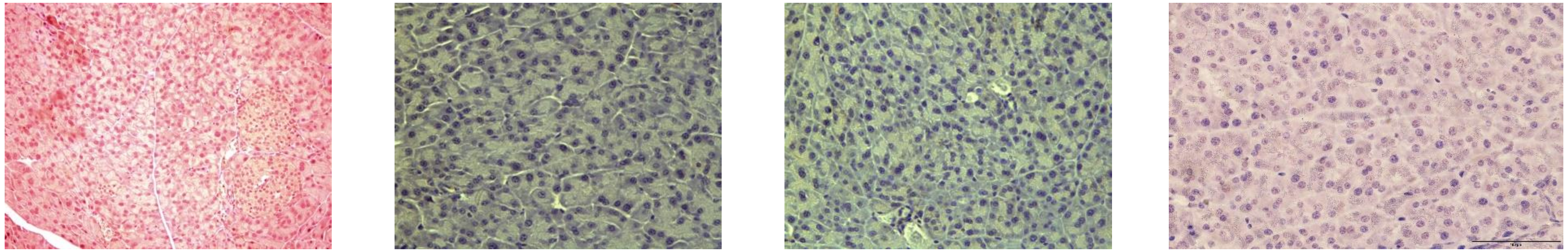

Additional represented photos for Figure 3G

MOUSE 1 MOUSE 2 MOUSE 3 MOUSE 4 MOUSE 5

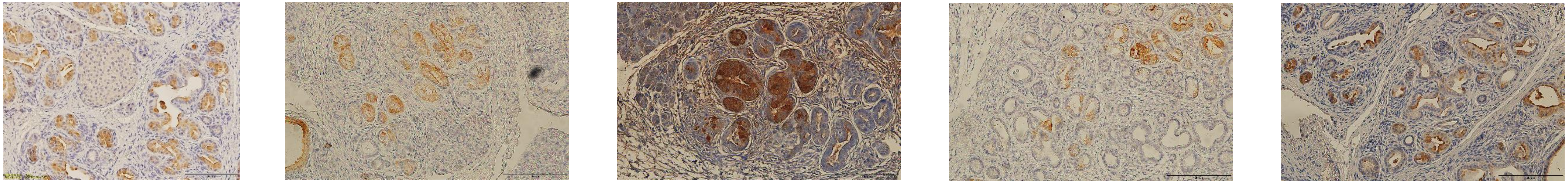

Additional represented photos for Figure 3H

MOUSE 1

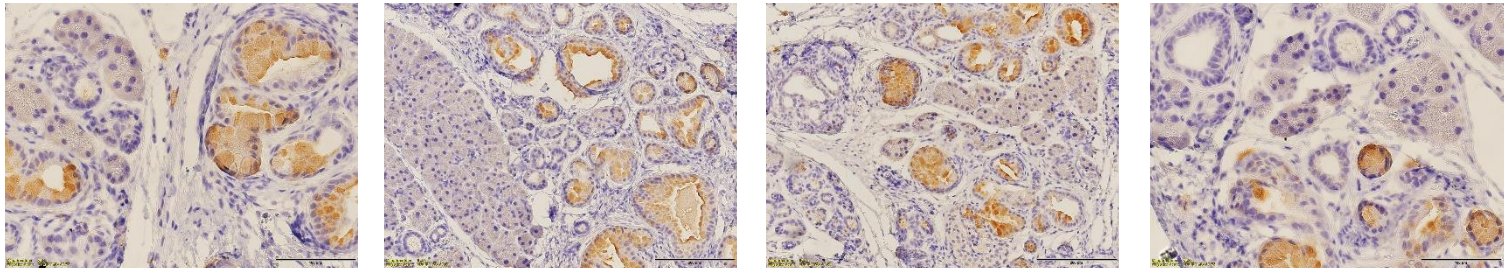

Additional represented photos for Figure 3I

MOUSE 1 MOUSE 2 MOUSE 3

Anti-KRT19

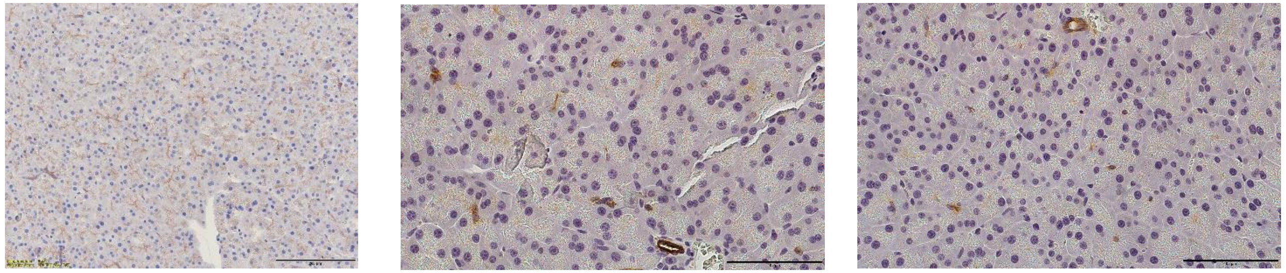

Additional represented photos for Figure 3J

MOUSE 1 MOUSE 2 MOUSE 3

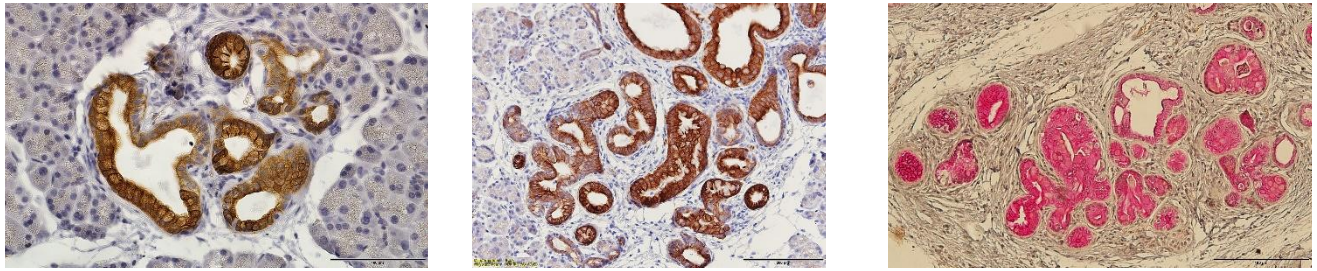

Additional represented photos for Figure 3K

MOUSE 1

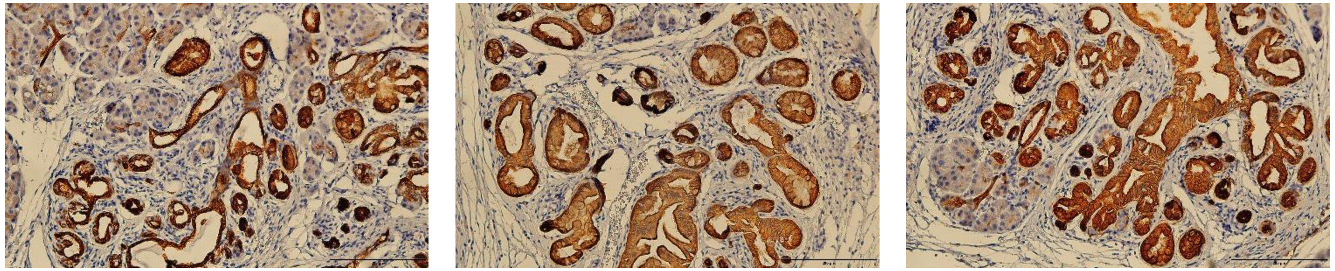

Anti-onecut2

Additional represented photos for Supplementary Figure 4k

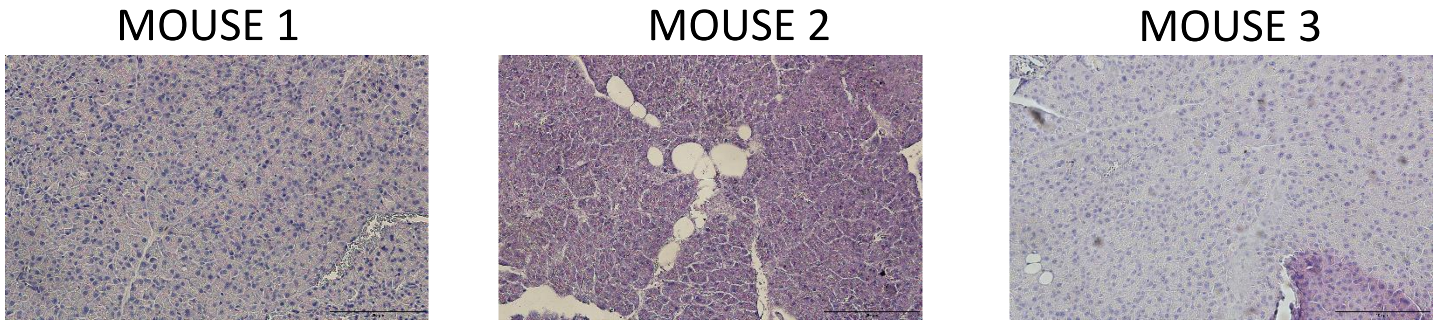

Additional represented photos for Supplementary Figure 4L

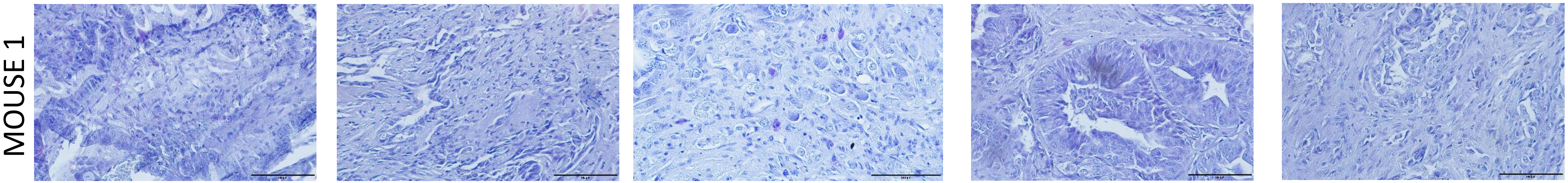

Additional represented photos for Supplementary Figure 4M

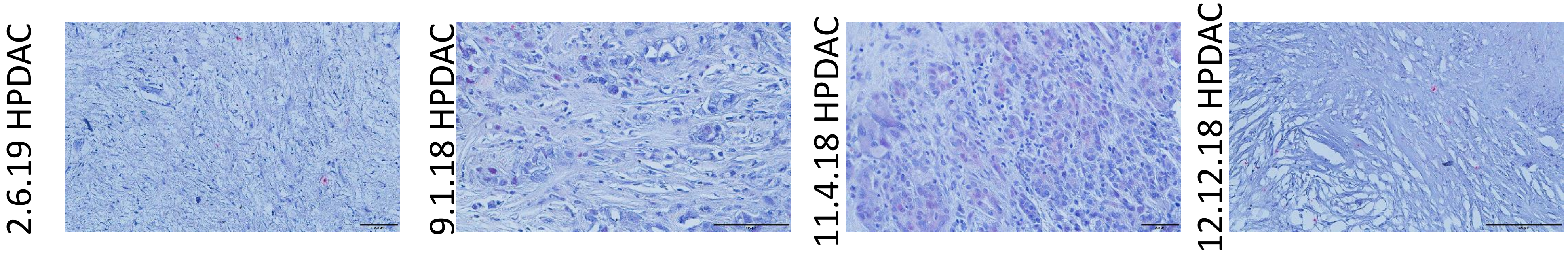

Anti-DCLK1 + Anti-Muc5ac

Additional represented photos for Figure 5A

MOUSE 1

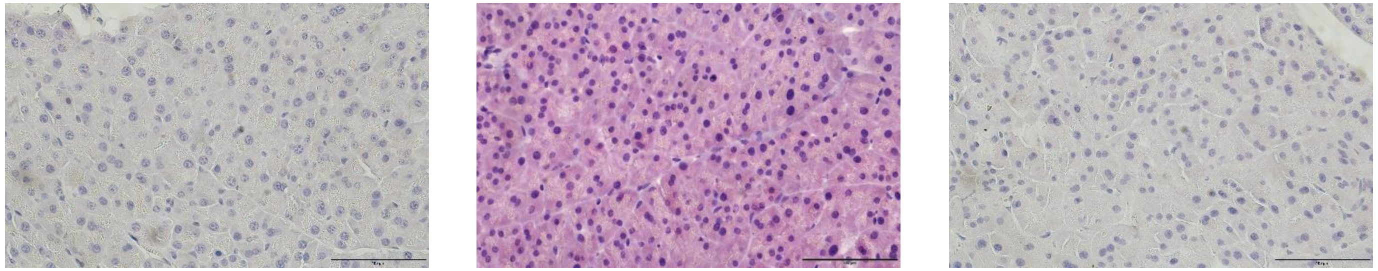

Additional represented photos for Figure 5B

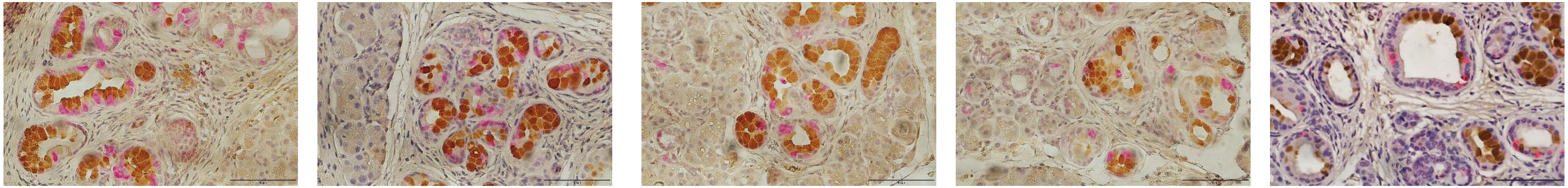

Additional represented photos for Figure 5C

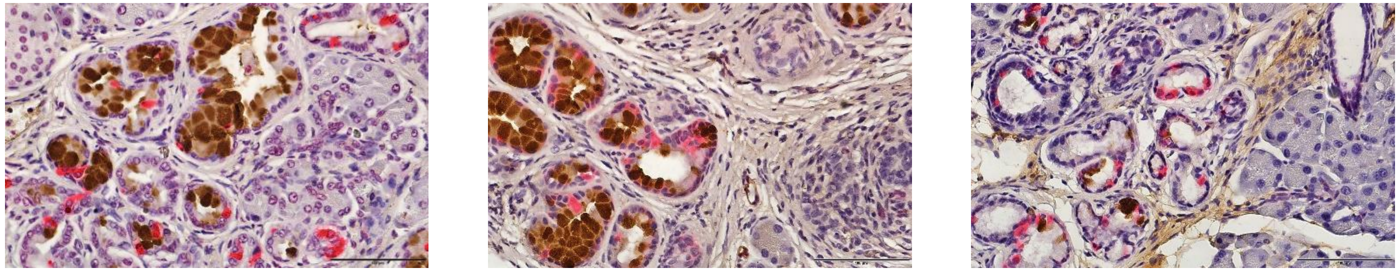

Anti-Tgfb1 + Anti-Muc5ac

Additional represented photos for Figure 5F

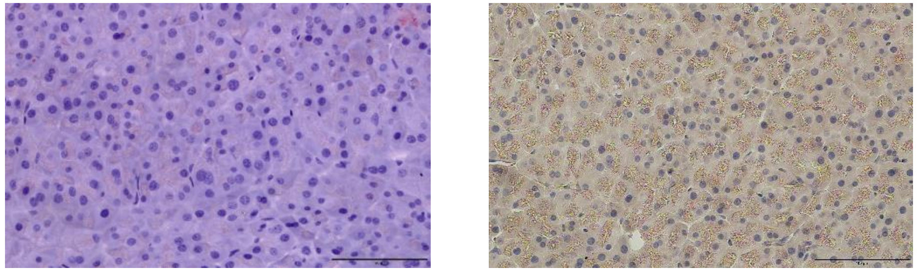

Additional represented photos for Figure 5I

MOUSE 1 MOUSE 2 MOUSE 3

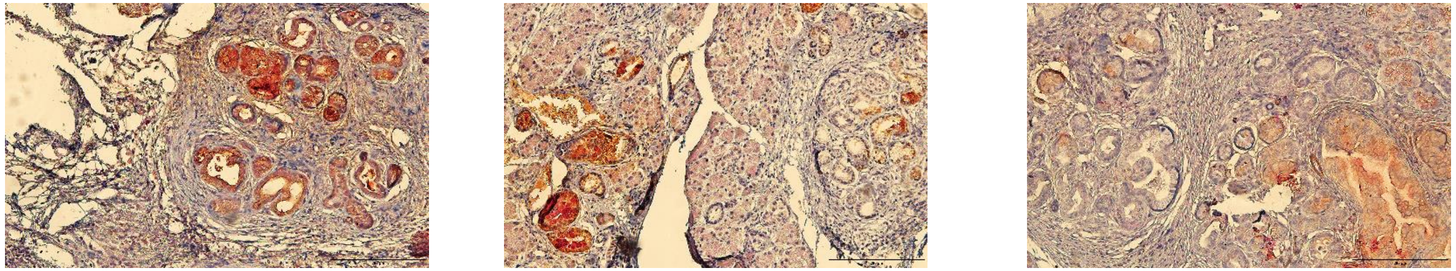

Additional represented photos for Supplementary Figure 7C-D

Anti-TFF1

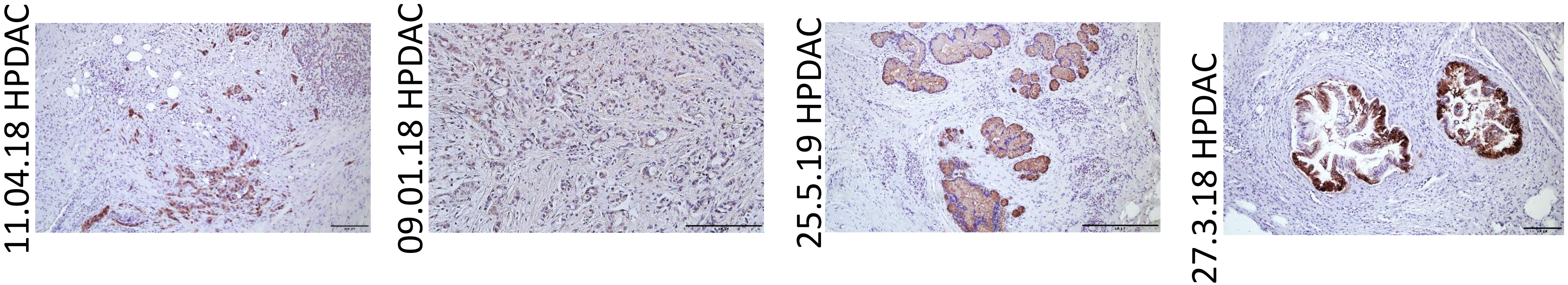

Additional represented photos for Supplementary Figure7E-F

Anti-Tgfb1

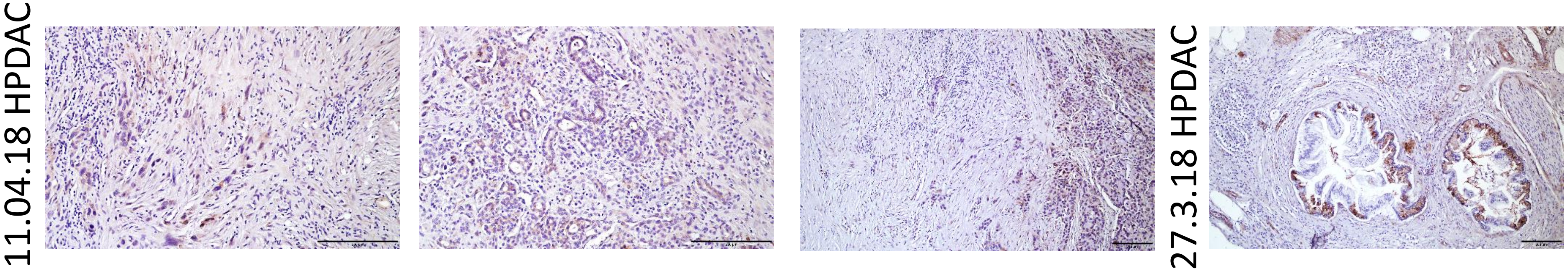

Additional represented photos for Supplementary Figure 8E-F

MOUSE 1 MOUSE 2 MOUSE 3

Anti-CD3 + Anti- Muc5ac

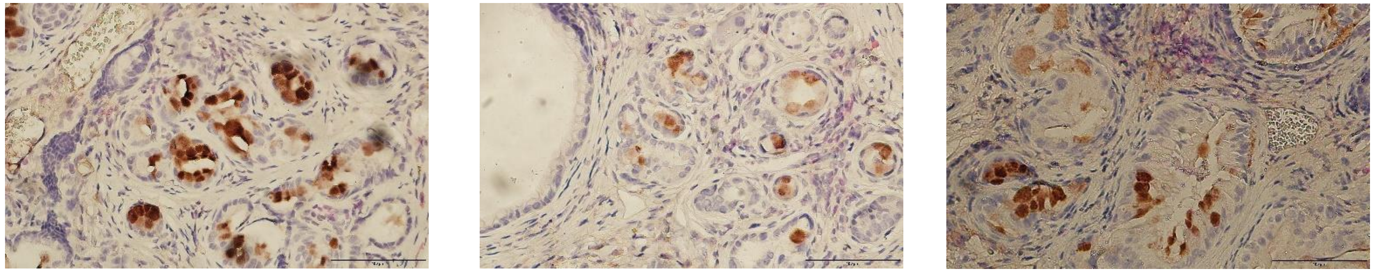

Additional represented photos for Supplementary Figure 8G-H

MOUSE 1 MOUSE 2 MOUSE 3

Anti-Ly6g + Anti- Muc5ac

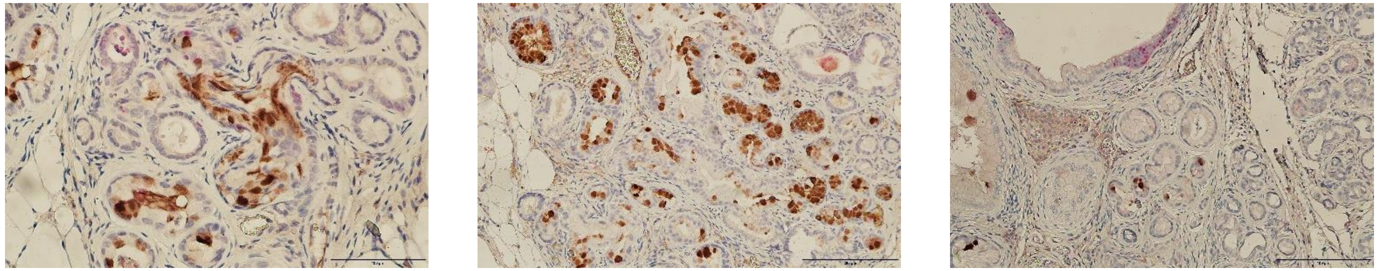

Additional represented photos for Supplementary Figure 8C-D

MOUSE 1 | MOUSE 2 | MOUSE 3

Anti-CD3

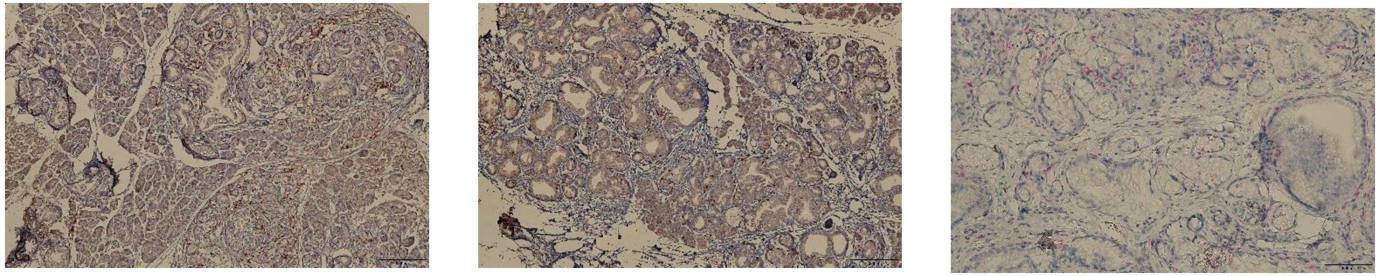

Additional represented photos for Supplementary Figure 8E-F

MOUSE 1 | MOUSE 2 | MOUSE 3

Anti-CD3 + Anti- Muc5ac

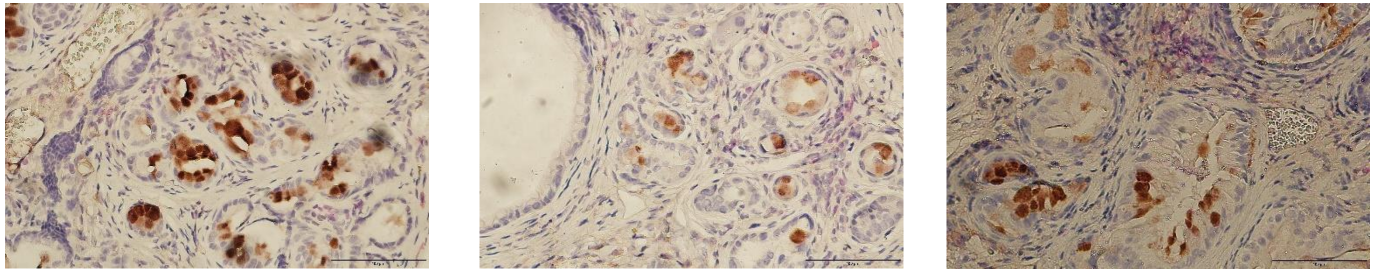

Additional represented photos for Supplementary Figure 8G-H

MOUSE 1 | MOUSE 2 | MOUSE 3

Anti-Ly6g + Anti- Muc5ac

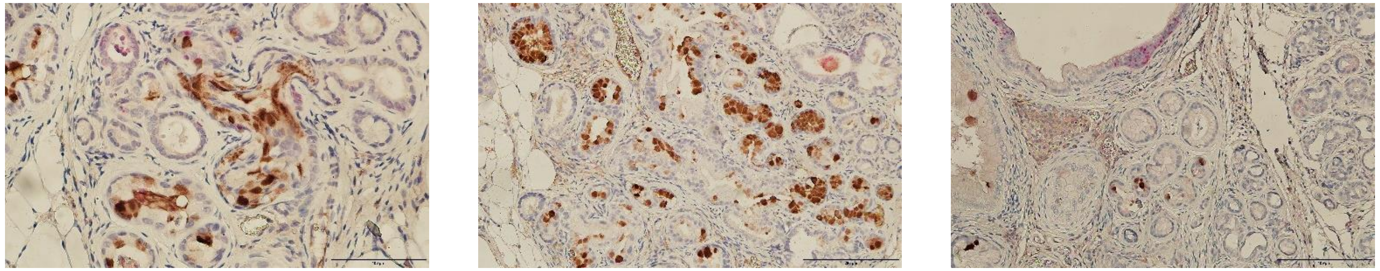

Microscopy images- quantification

Anti-DCLK1

MOUSE#1

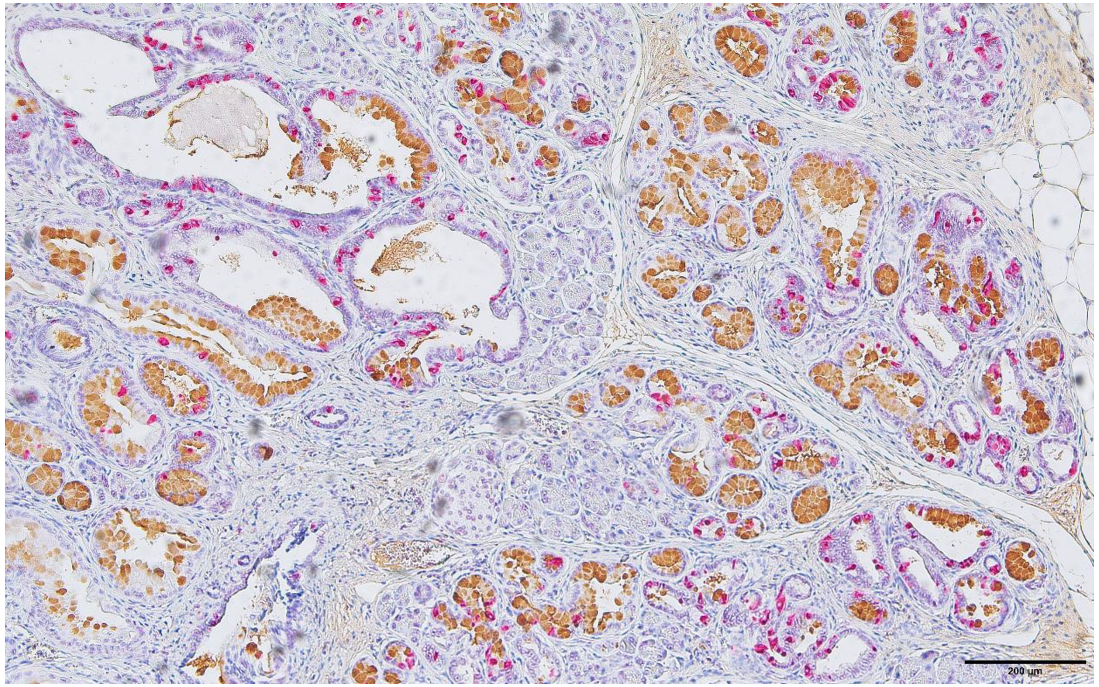

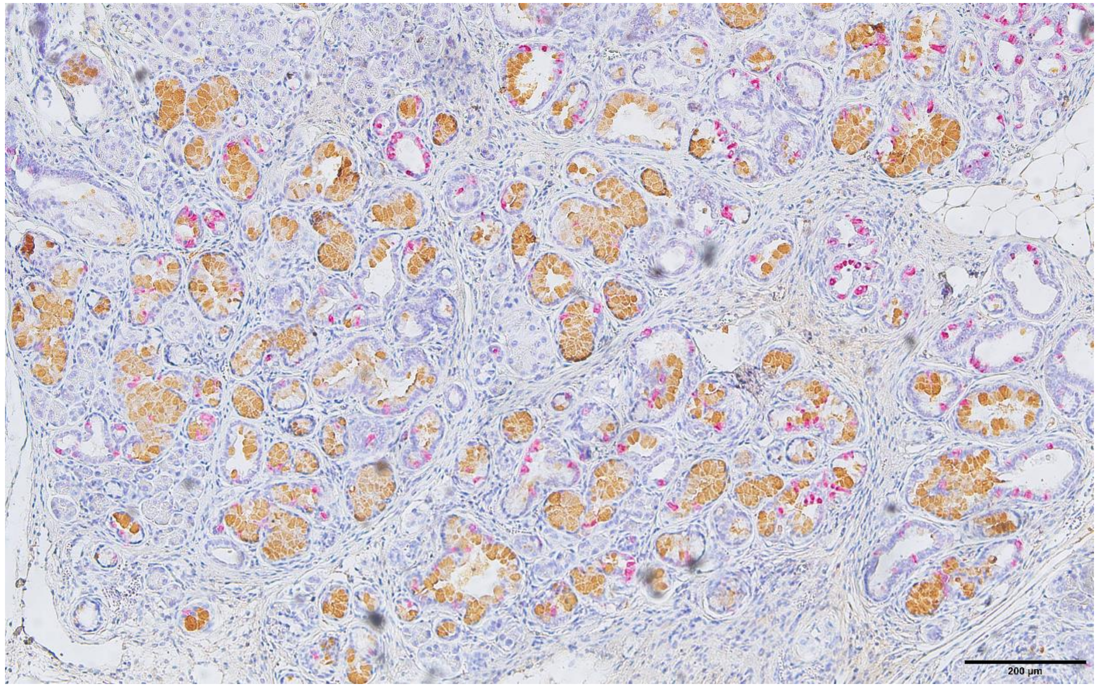

Additional represented photos for Figure 4E

Anti-DCLK1

MOUSE#2

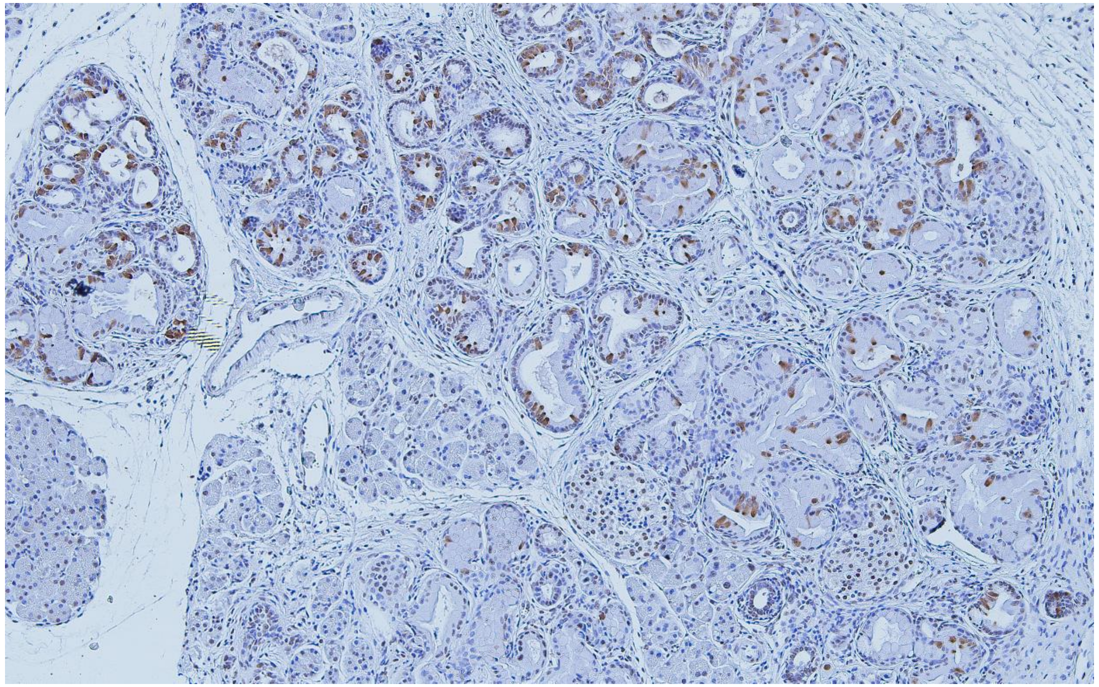

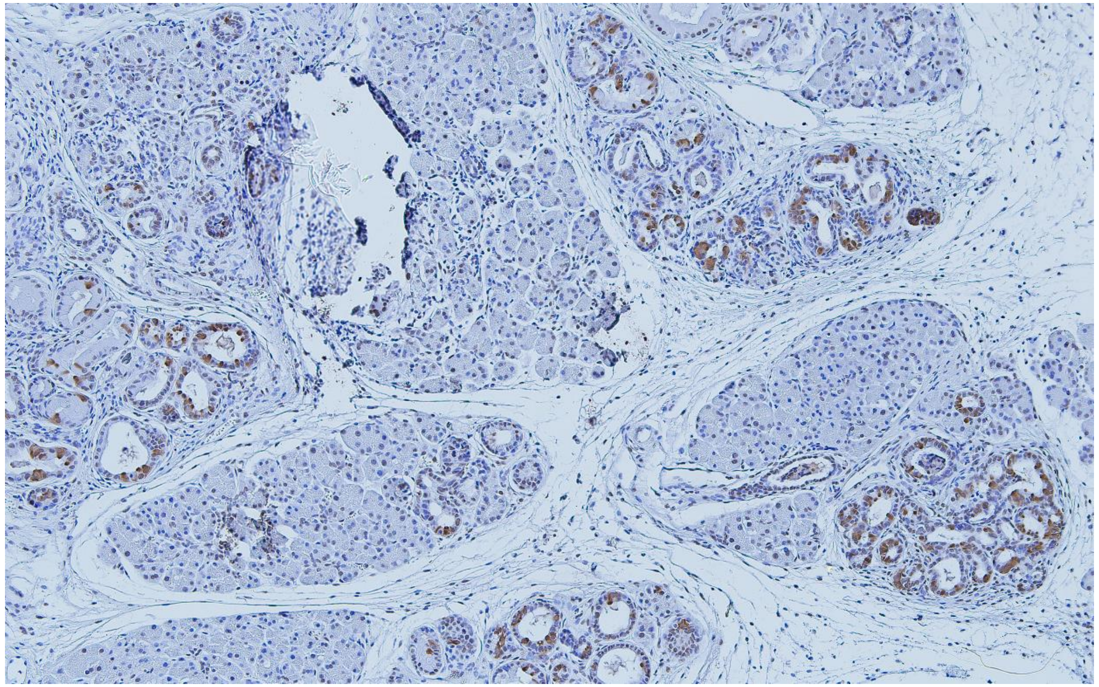

Additional represented photos for Figure 4E

Anti-DCLK1

MOUSE#3

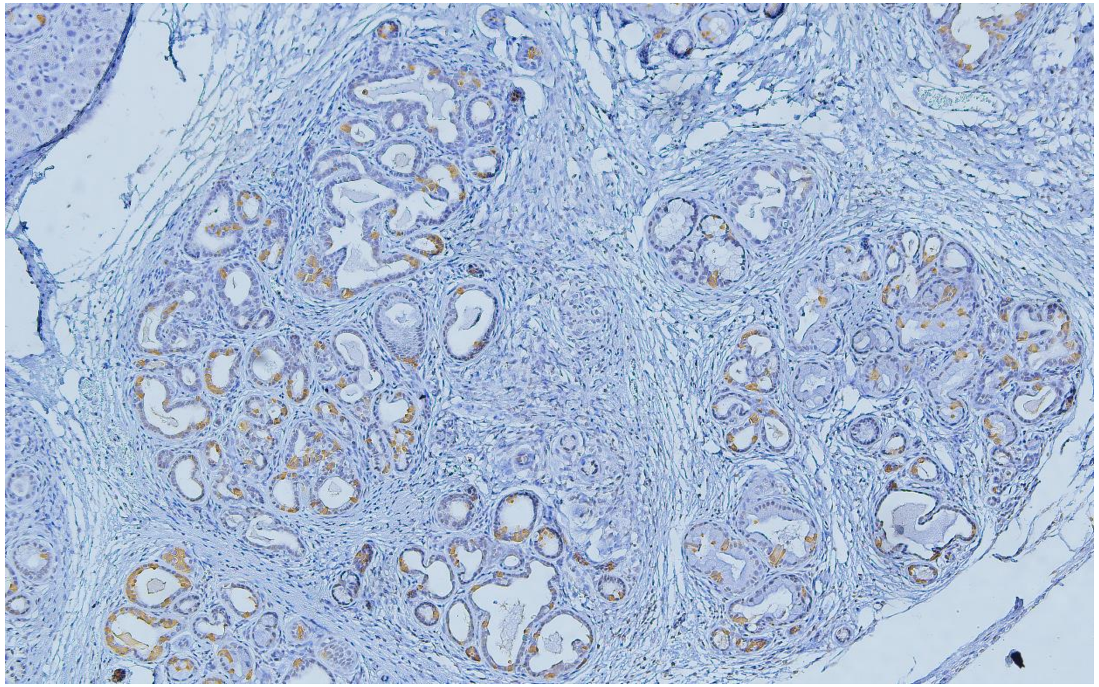

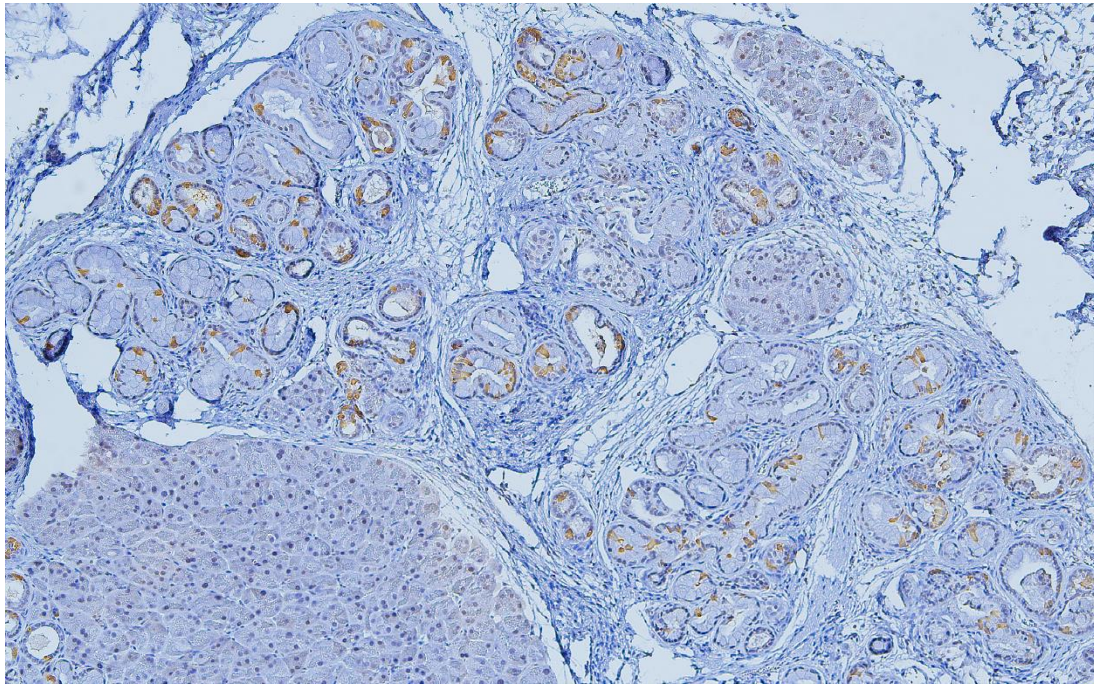

Additional represented photos for Figure 4E

Anti-TFF1

MOUSE#1

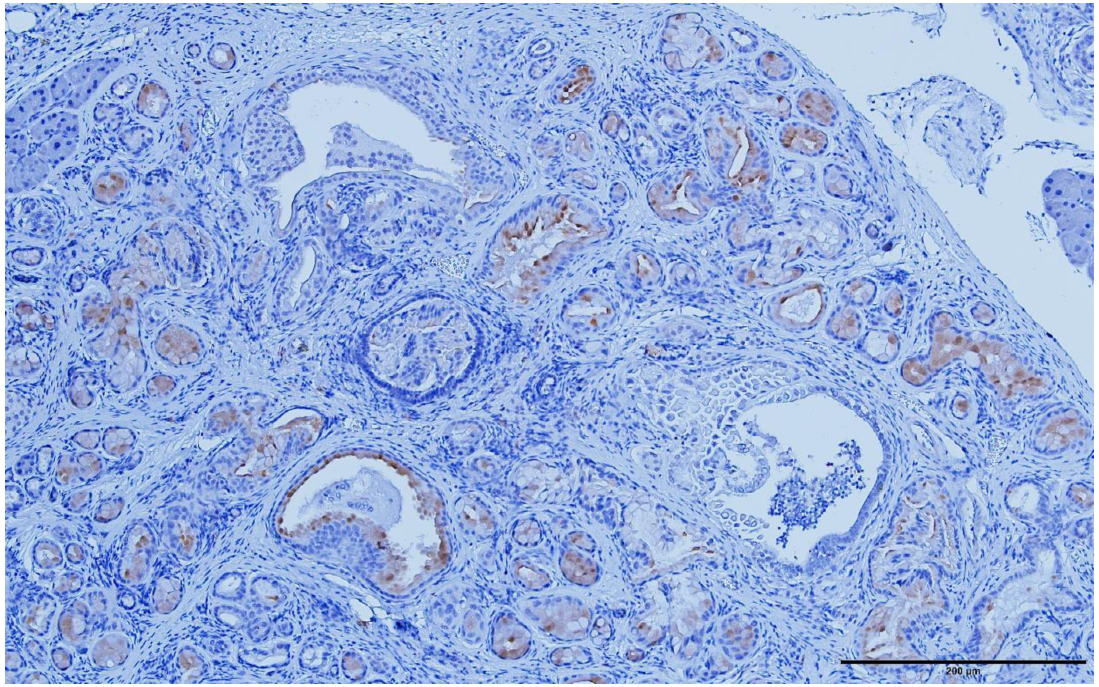

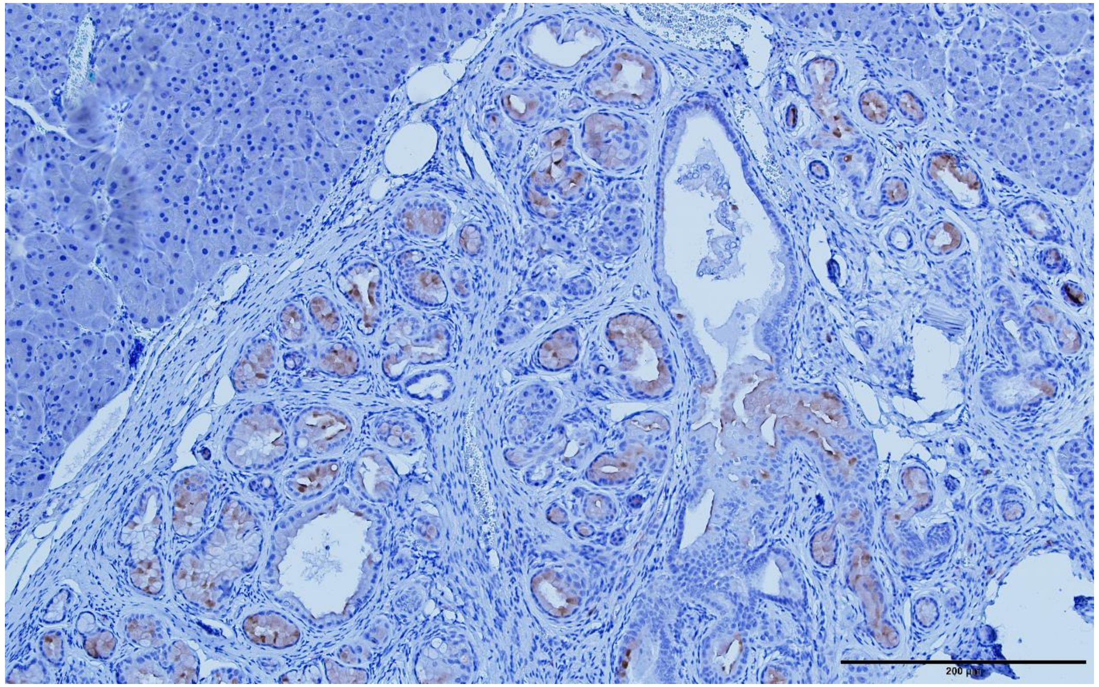

Additional represented photos for Figure 4N

Anti-TFF1

MOUSE#2

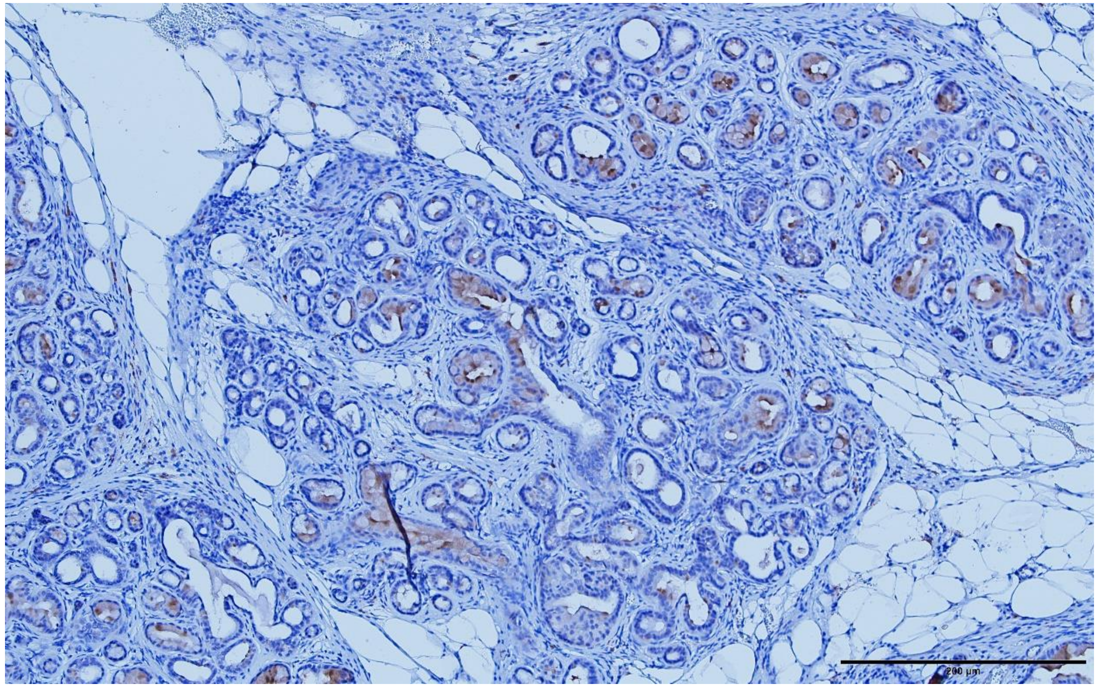

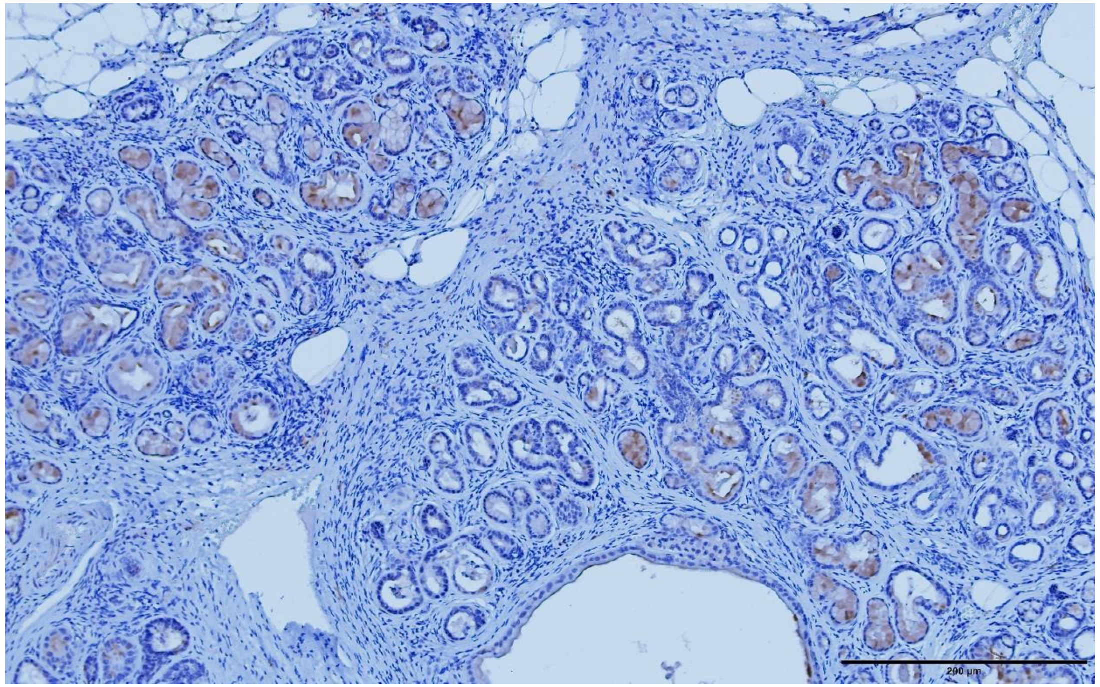

Additional represented photos for Figure 4N

Anti-TFF1

MOUSE#3

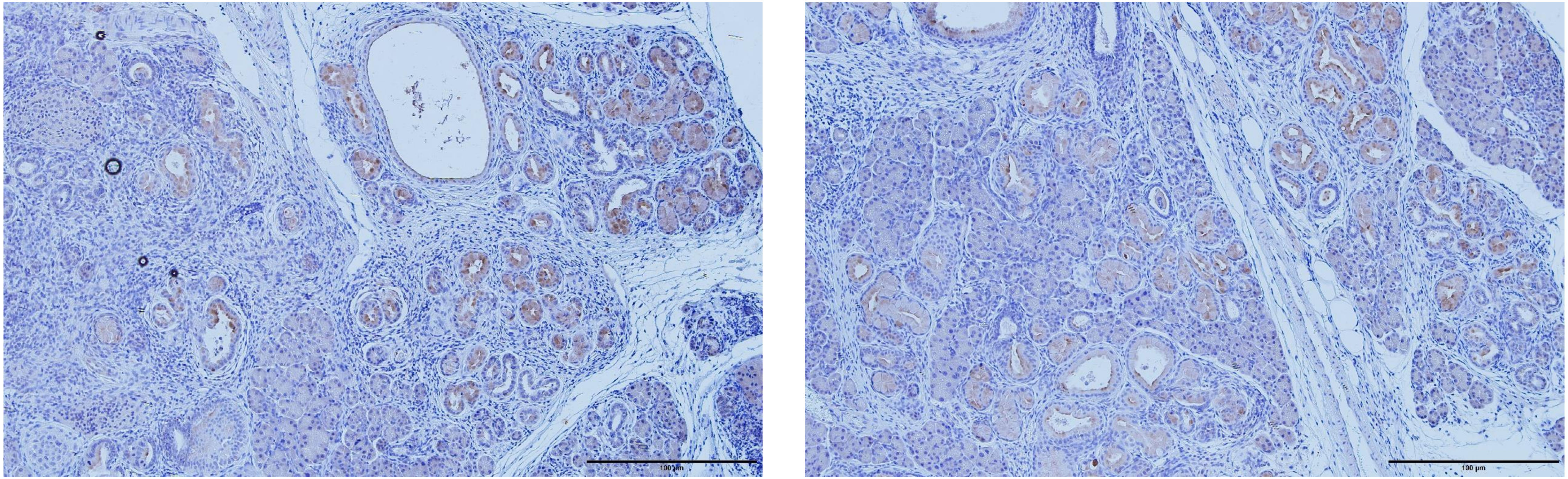

Additional represented photos for Figure 4N

Anti-MKi67

MOUSE#1

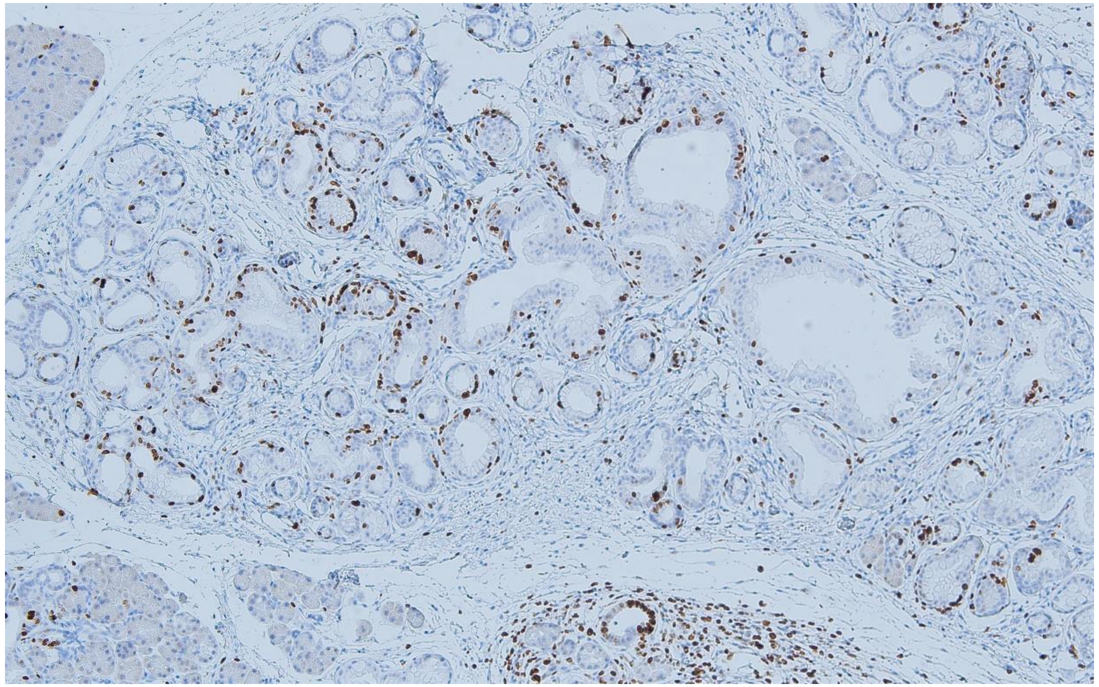

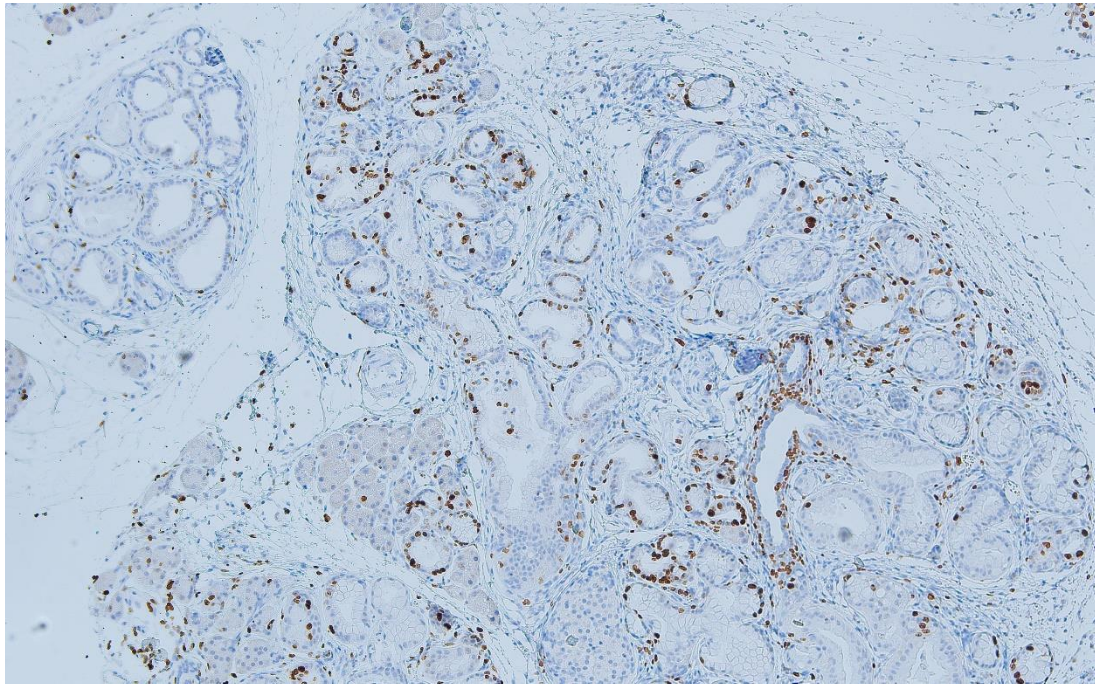

Additional represented photos for Figure 4P

Anti-MKi67

MOUSE#2

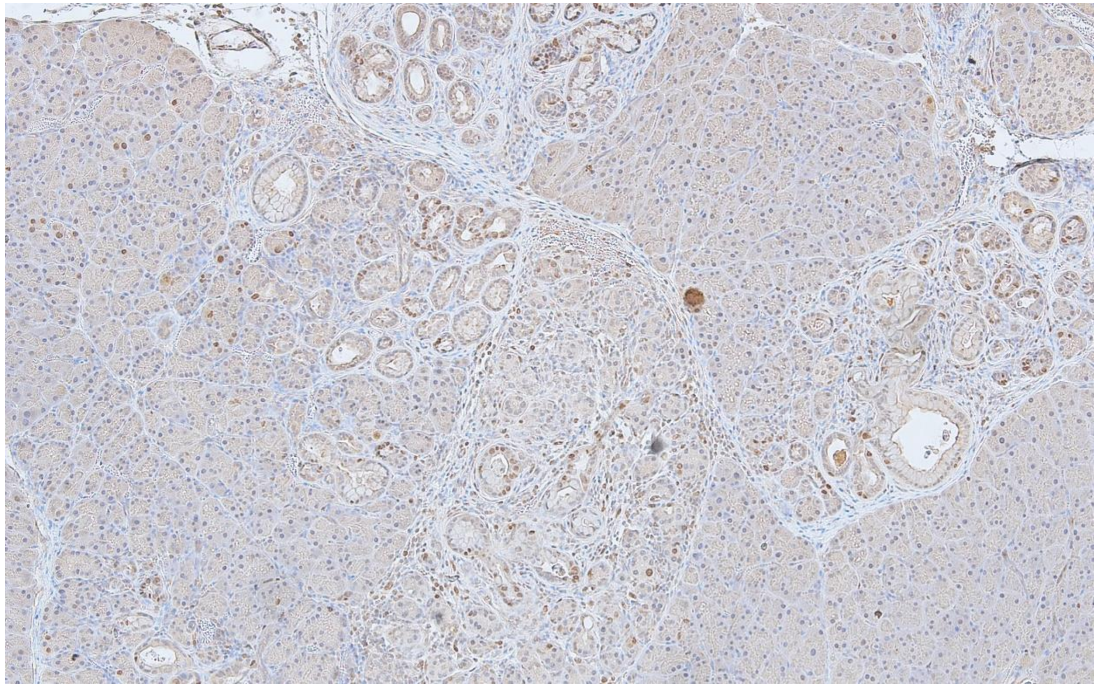

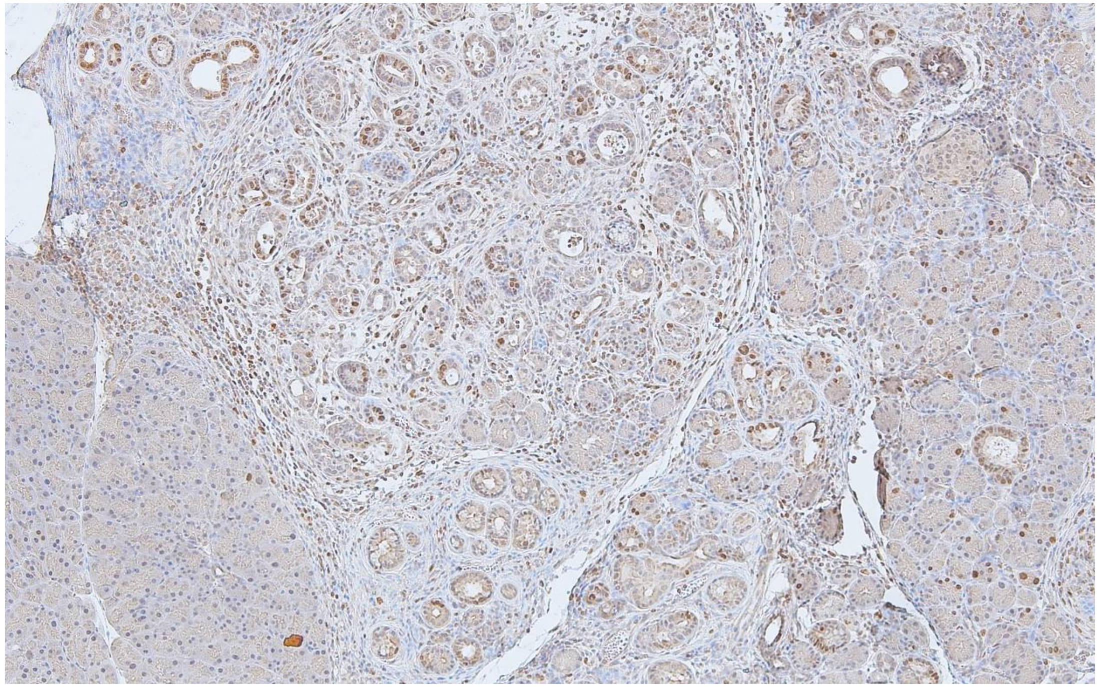

Additional represented photos for Figure 4P

Anti-MKi67

MOUSE#3

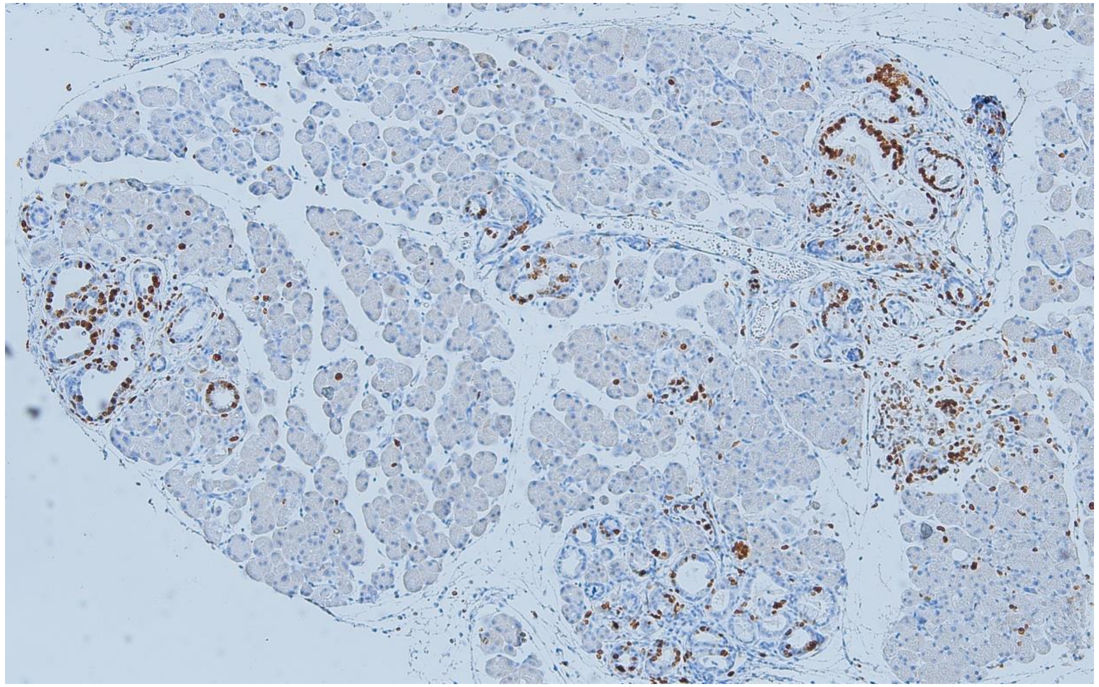

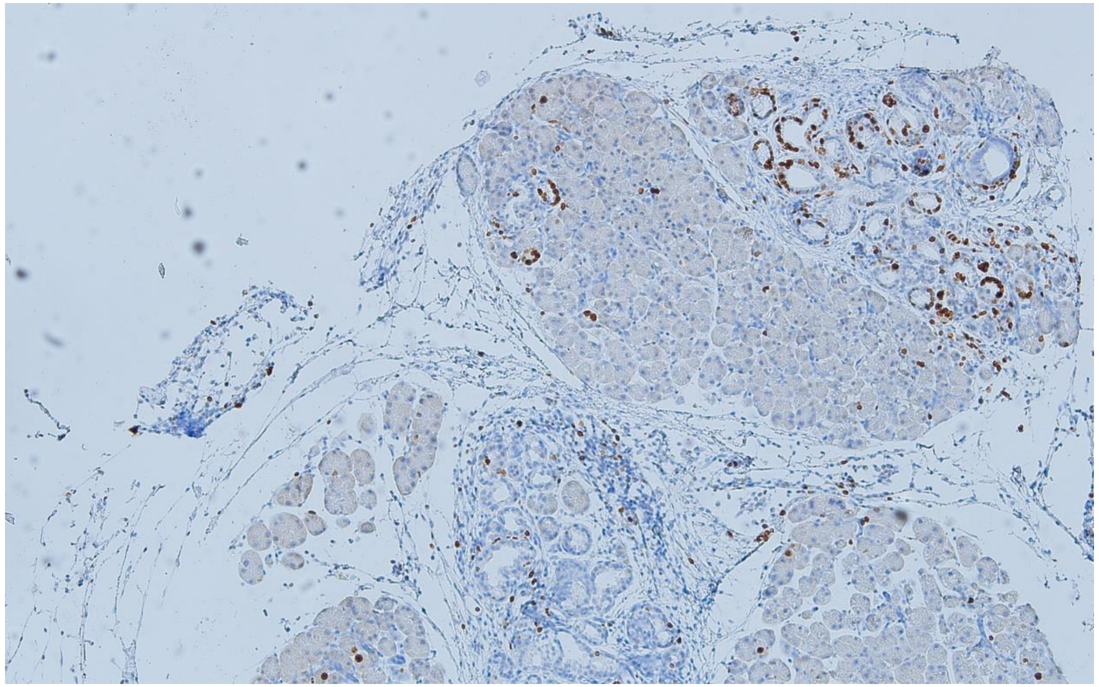

Additional represented photos for Figure 4P

Anti-TGFb1

MOUSE#1

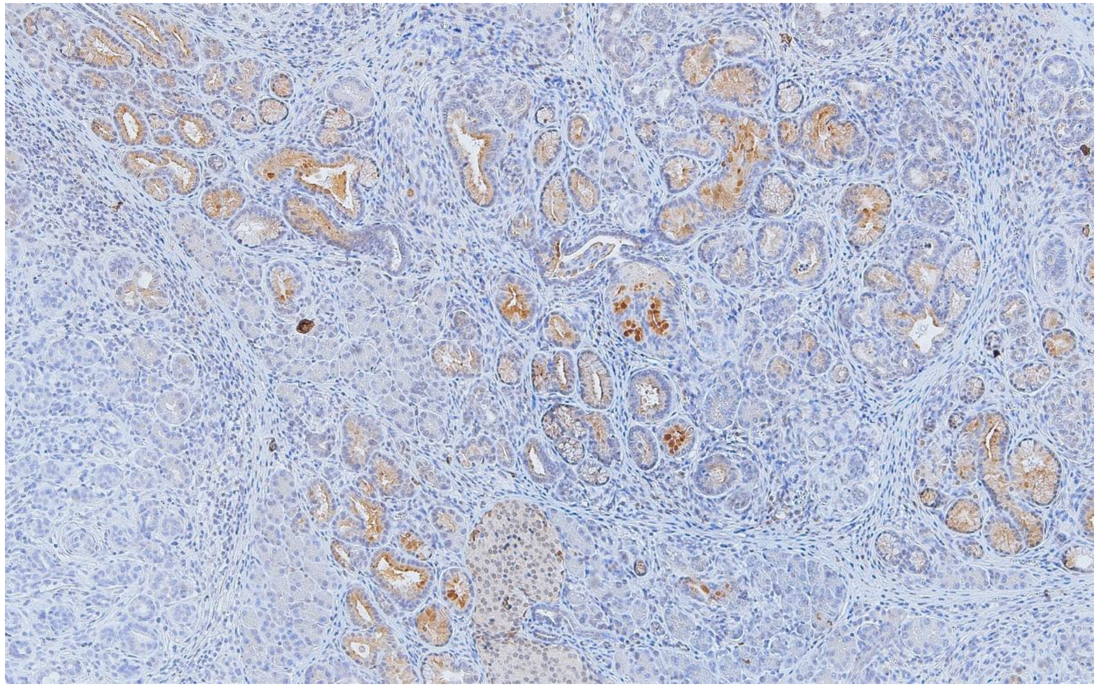

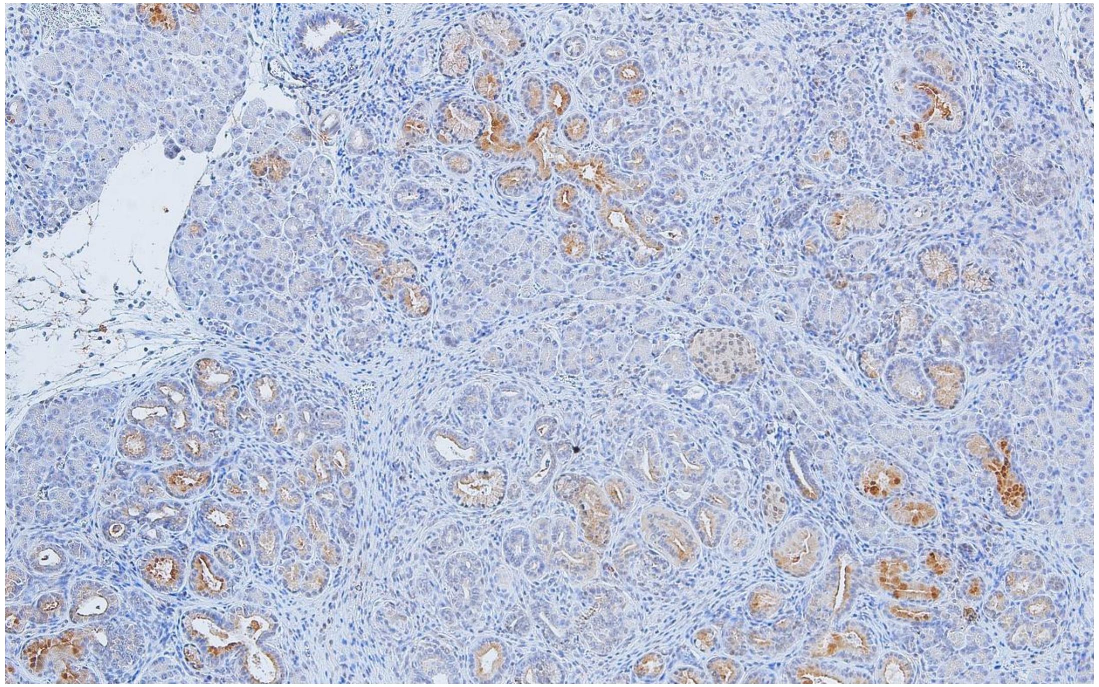

Additional represented photos for Figure 4G

Anti-TGFb1

MOUSE#2

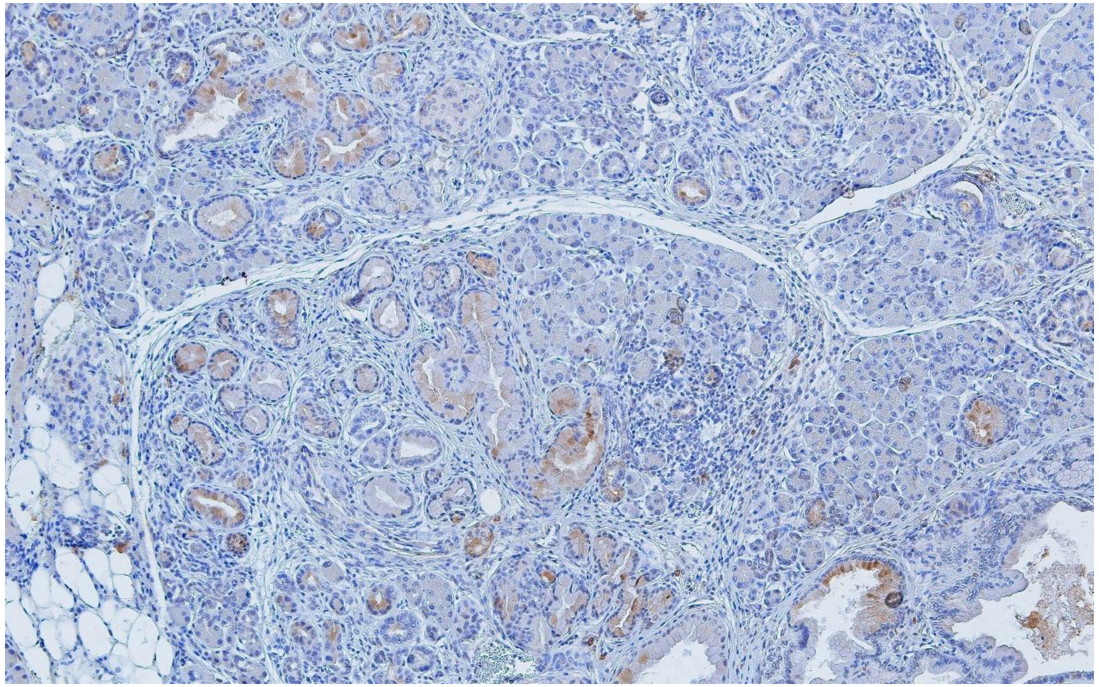

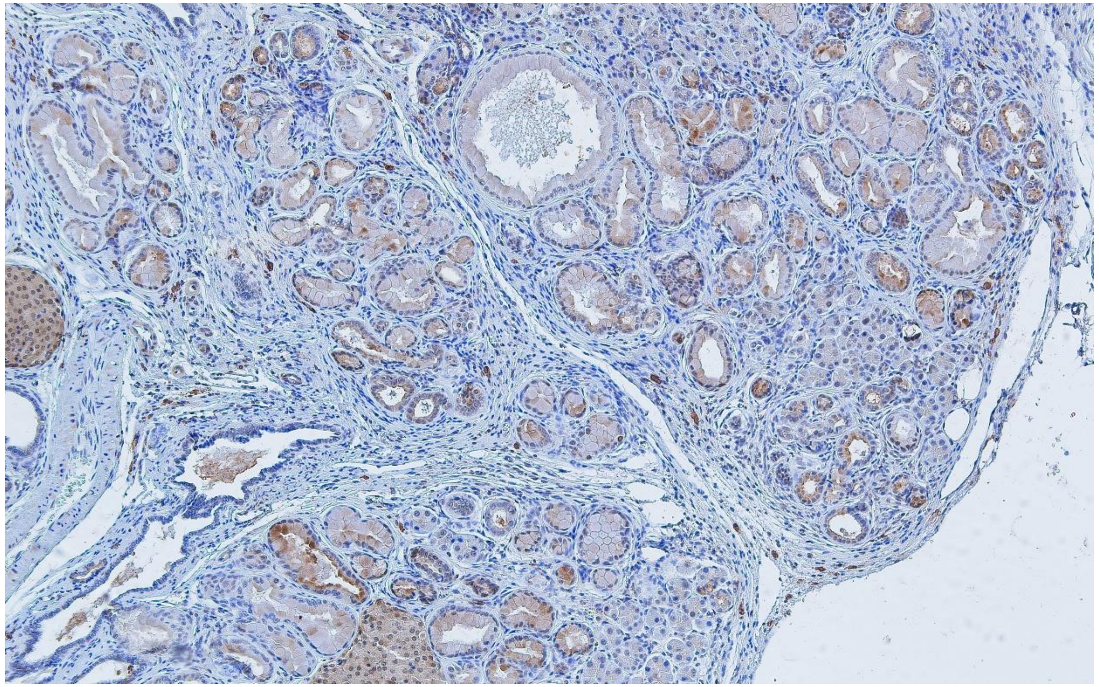

Additional represented photos for Figure 5

Additional represented photos for Figure 4G

Anti-TGFb1

MOUSE#3

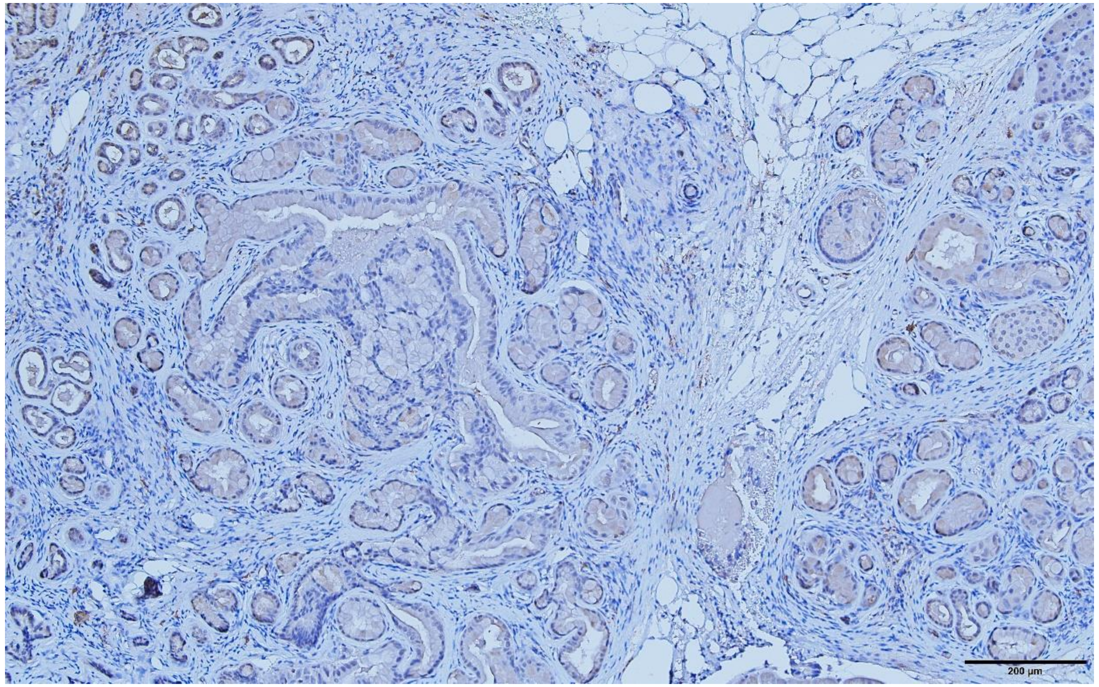

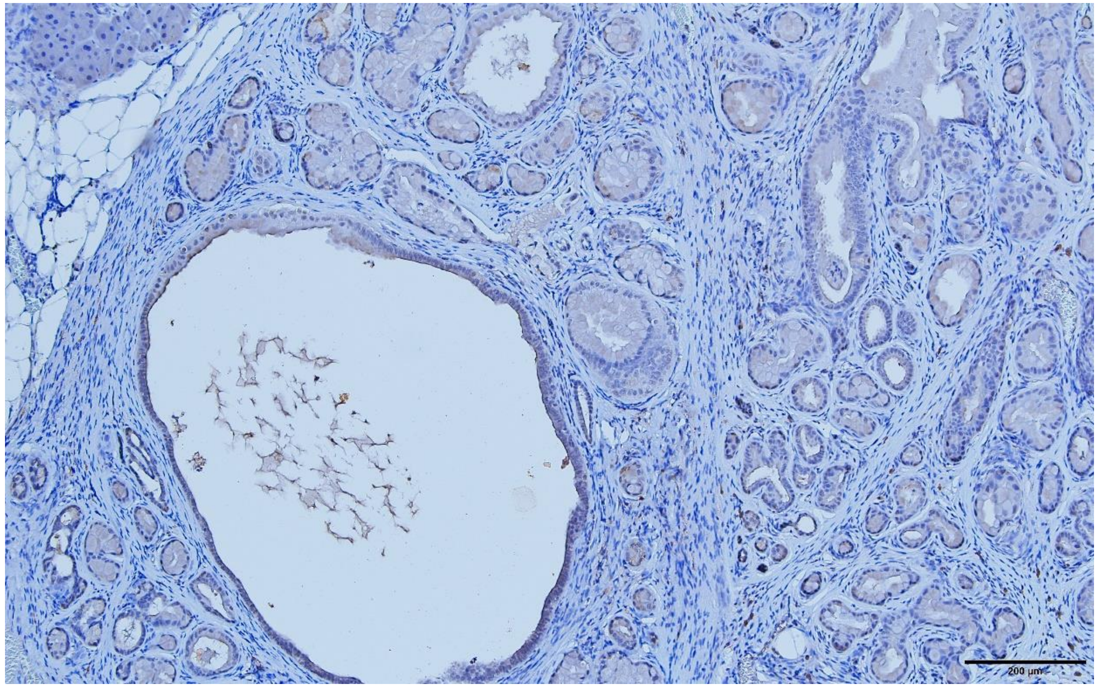

Additional represented photos for Figure 5
Additional represented photos for Figure 4G

Anti-Ins1

MOUSE#1

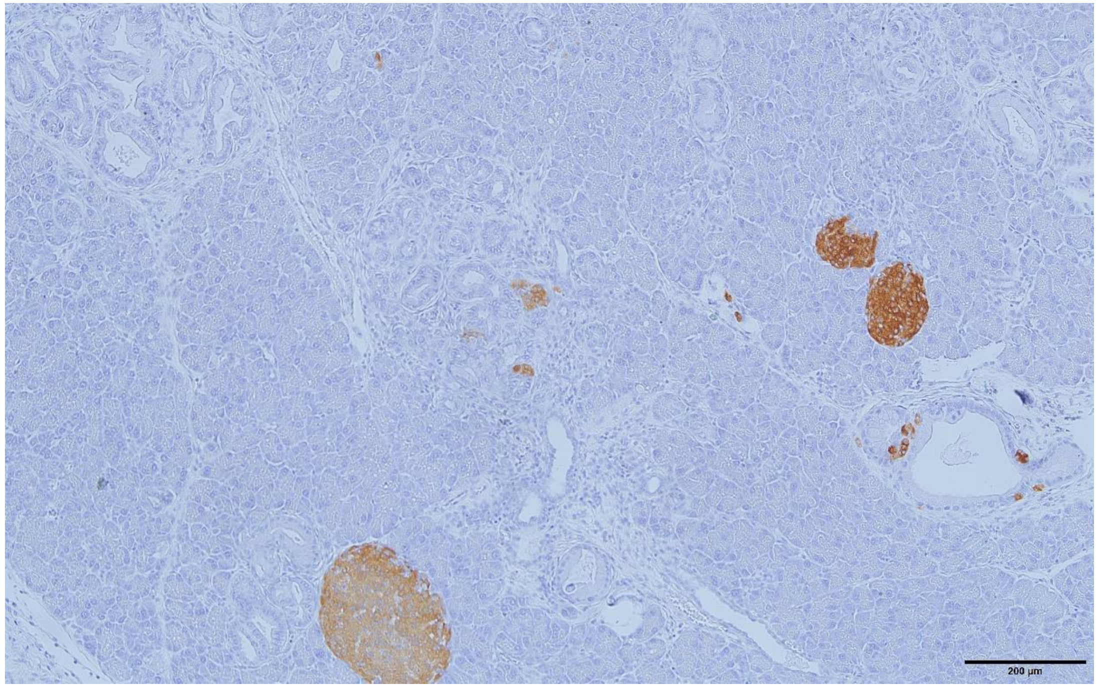

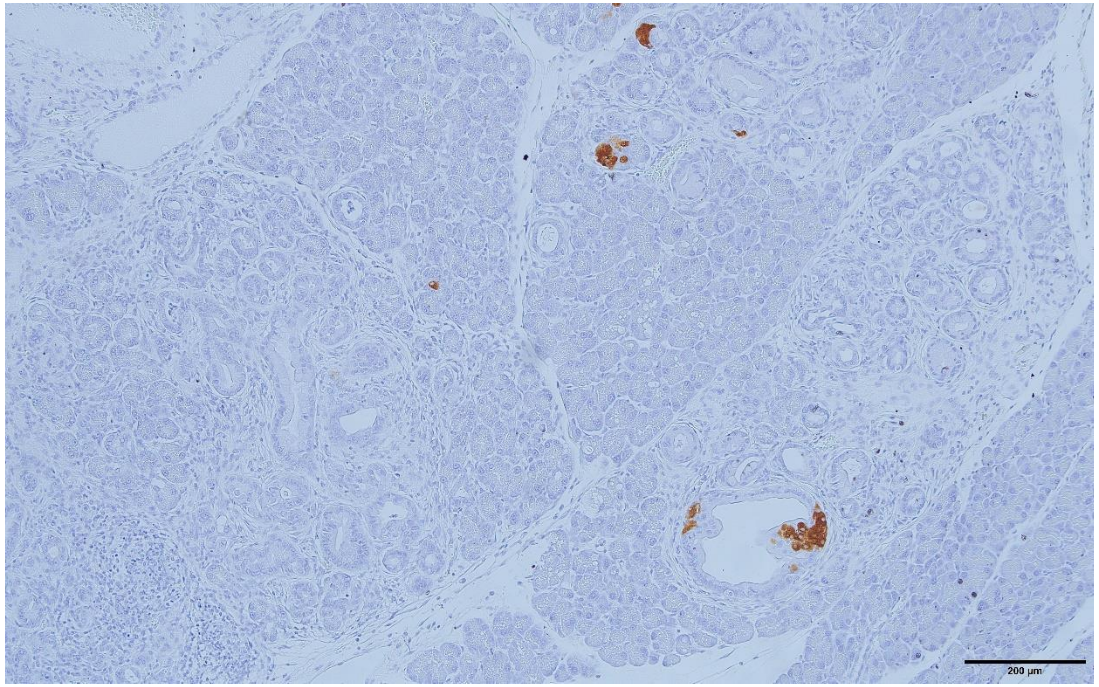

Additional represented photos for Figure 4B

Anti-Ins1

MOUSE#2

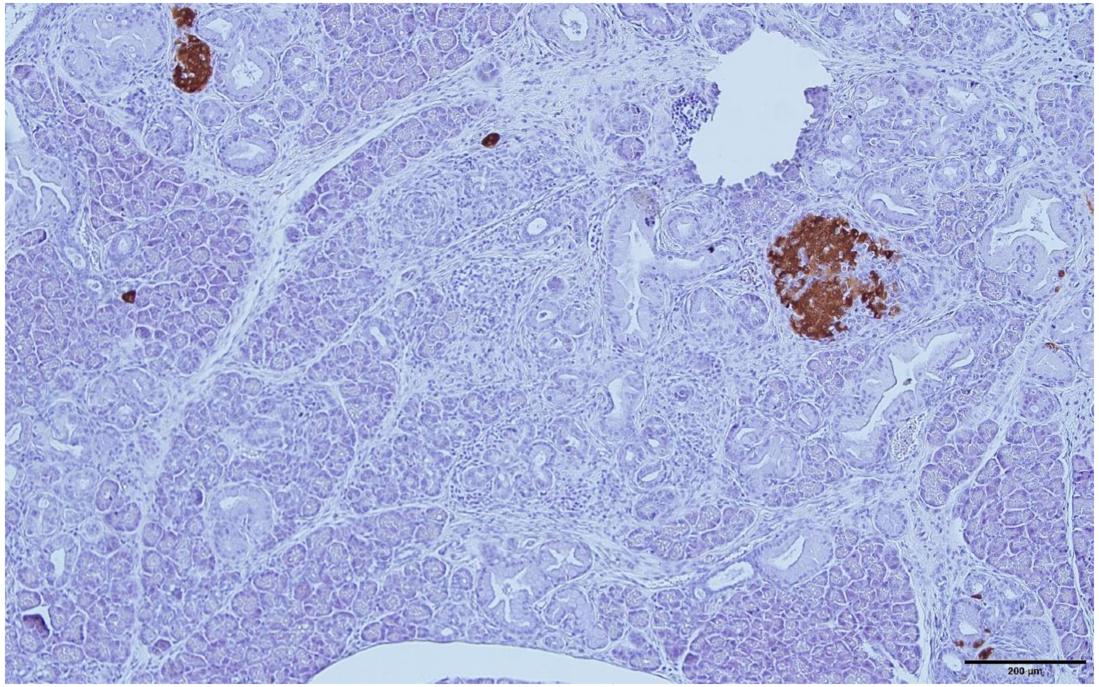

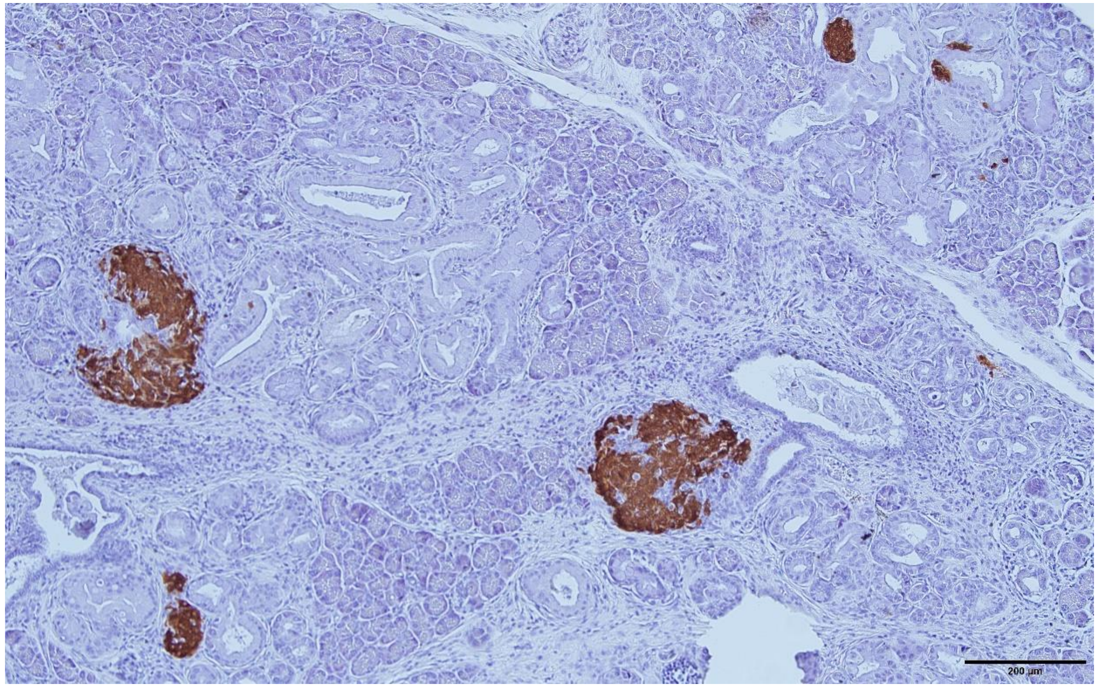

Additional represented photos for Figure 4B

Anti-Ins1

MOUSE#3

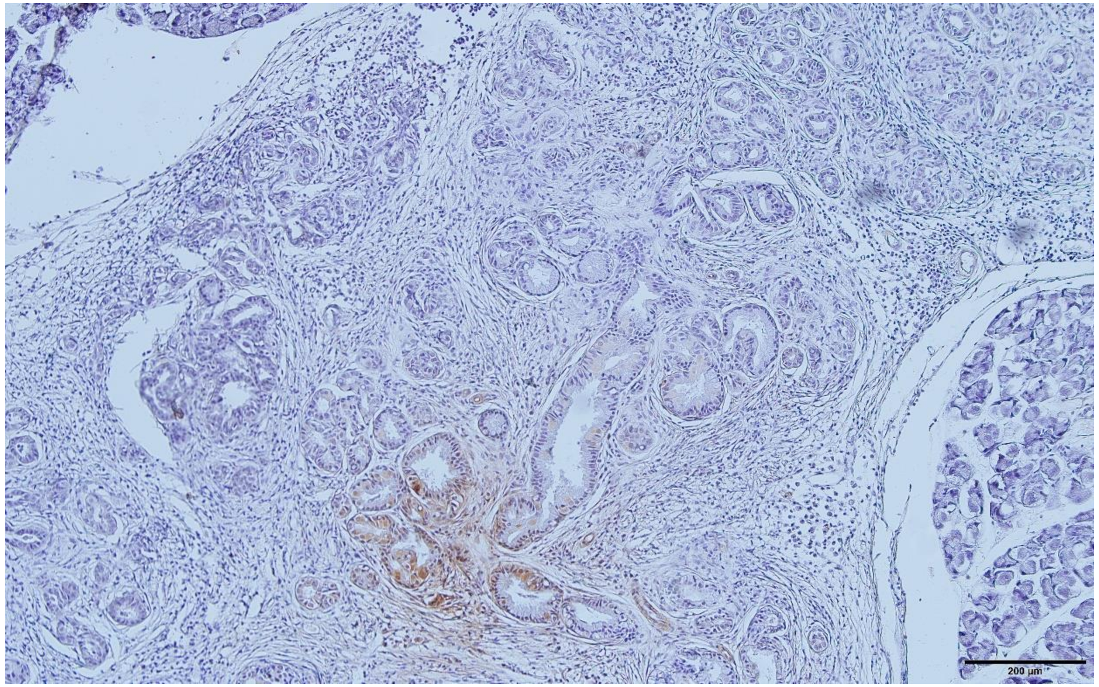

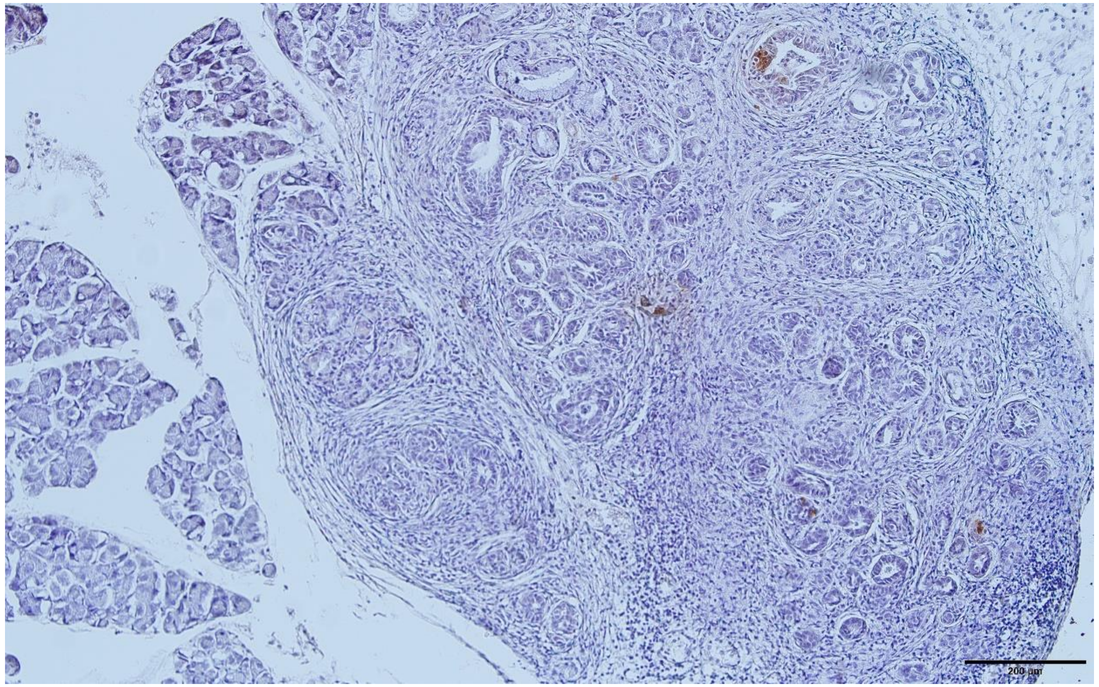

Additional represented photos for Figure 4B

Supplementary Figure 1a

Image Number	1#	2#	3#	4#	5#	6#	average	stdev
PT	0	0	0	0	0	0	0	0
17 days	0	0	0	0	0	2	0.3	0.8
1.5 months	0	0	0	0	1	12	2.1	4.8
3 months	43	30	54	61	35	21	40.6	15.0
5 months	47	58	66	57	58	44	55	8.0
9 months	93	83	66	59	84	98	80.5	15.1
15 months	103	89	123	96	91	84	97.6	13.9

Batch Analysis Report

Run Date: 7/26/20 11:00 AM
Experiment: 06/11/2018 2
User ID: Administrator
Statistics Output: N/A
Worksheet PDF Output: E:\SORTER\2018 2-Batch_Analysis_26072020105943.pdf

Specimen_001

Tube	Status	Run Time
spleen	OK	7/26/20 11:00 AM
6 month prt	OK	7/26/20 11:00 AM
6 month prt_001	OK	7/26/20 11:00 AM

BD FACSDiva 8.0.1

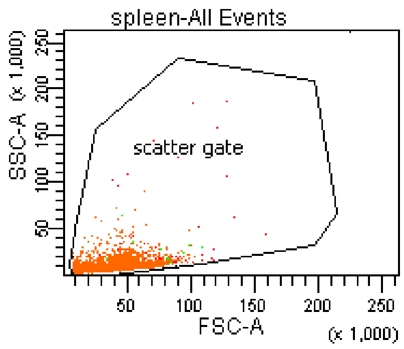

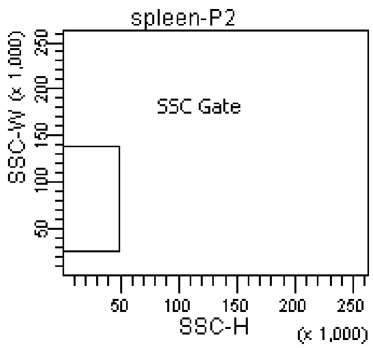

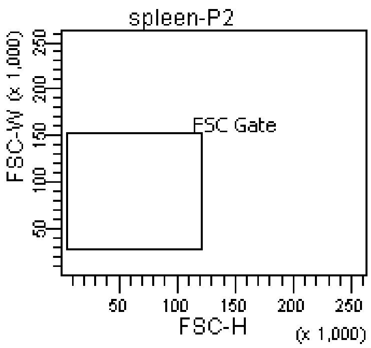

Tube: spleen

Population	#Events	%Parent	%Total
All Events	10,000	####	100.0
scatter gate	9,768	97.7	97.7
SSC Gate	9,641	98.7	96.4
FSC Gate	9,613	99.7	96.1
P1	9,613	100.0	96.1
CD45+	####	####	####
T+	0	0.0	0.0
P2	0	0.0	0.0

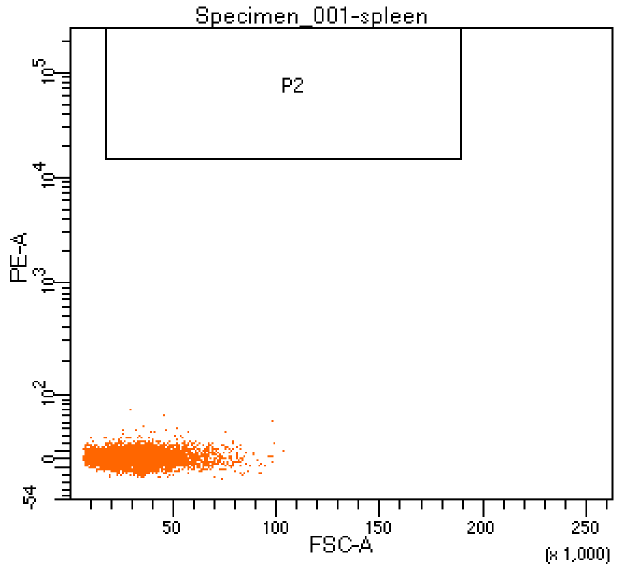

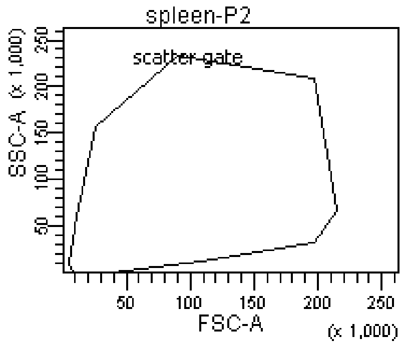

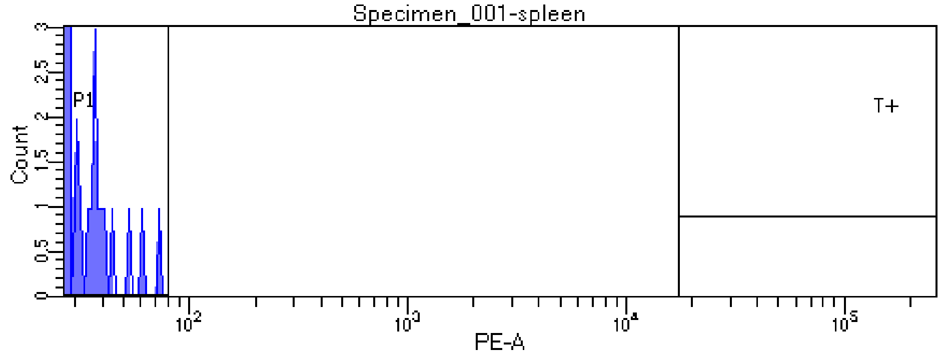

BD FACSDiva 8.0.1

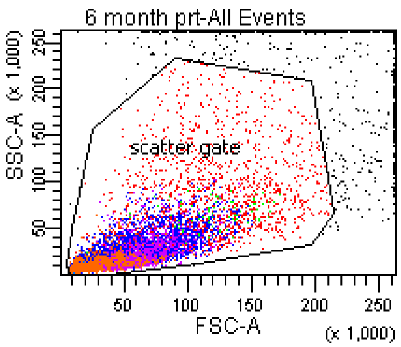

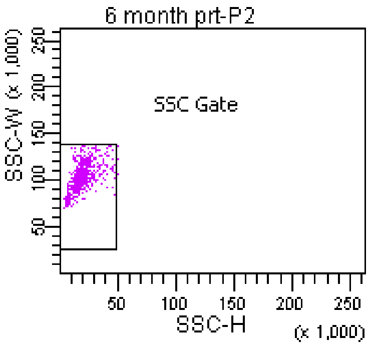

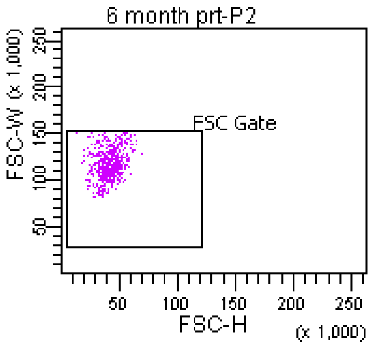

Tube: 6 month prt

Population	#Events	%Parent	%Total
All Events	10,000	####	100.0
scatter gate	8,904	89.0	89.0
SSC Gate	7,309	82.1	73.1
FSC Gate	7,179	98.2	71.8
P1	3,428	47.8	34.3
CD45+	####	####	####
T+	548	7.6	5.5
P2	562	7.8	5.6

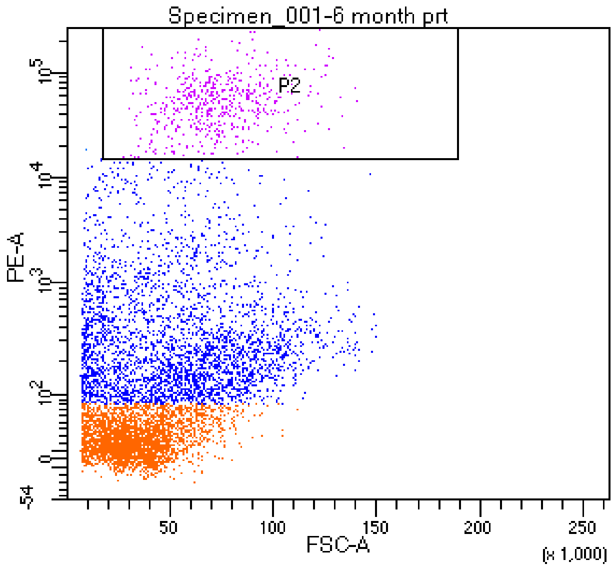

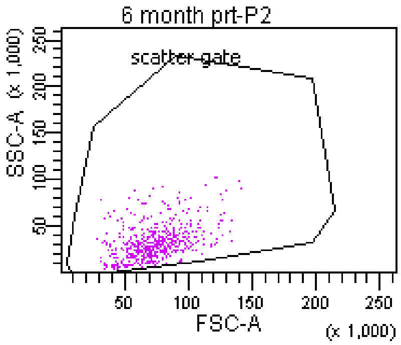

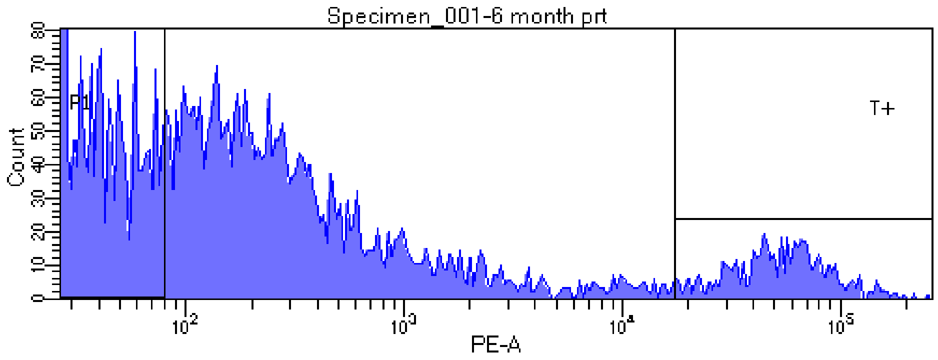

BD FACSDiva 8.0.1

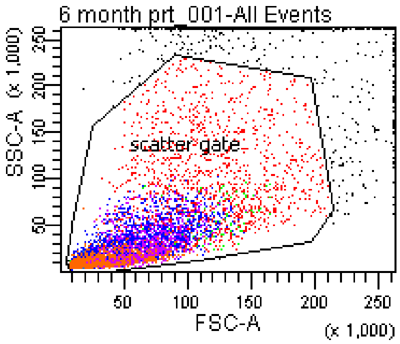

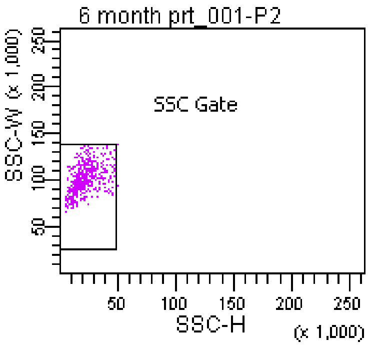

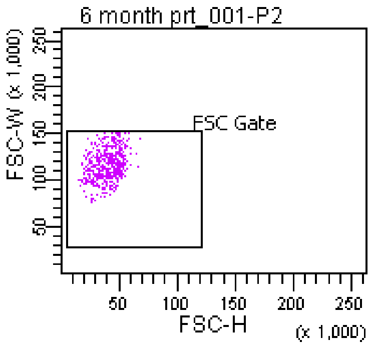

Tube: 6 month prt_001

Population	#Events	%Parent	%Total
All Events	7,020	####	100.0
scatter gate	6,132	87.4	87.4
SSC Gate	4,790	78.1	68.2
FSC Gate	4,693	98.0	66.9
P1	2,048	43.6	29.2
CD45+	####	####	####
T+	510	10.9	7.3
P2	522	11.1	7.4

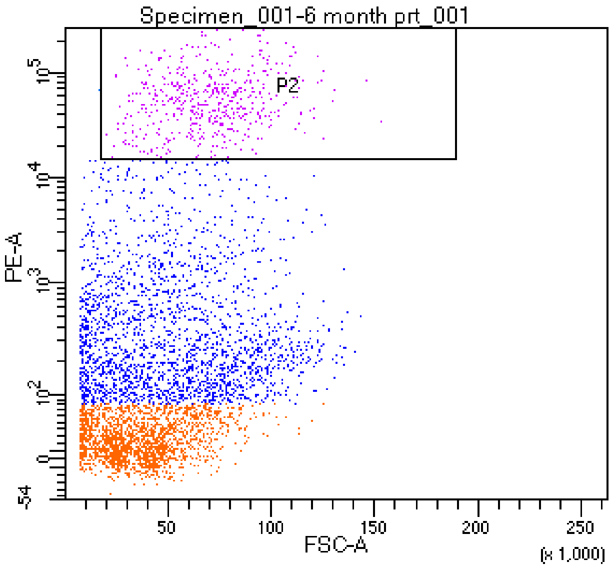

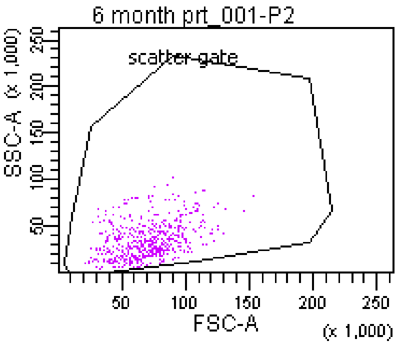

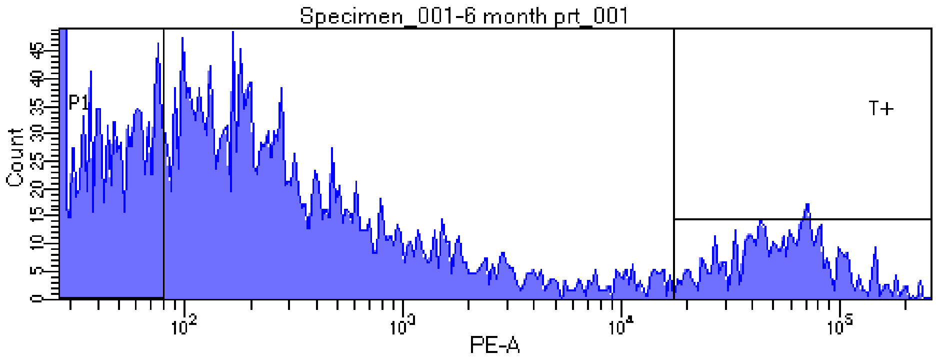

Supplement: Supplementary file 15 — Source Data [file 41467_2020_18207_MOESM15_ESM.pdf]
